# Supplementary figures and images for: A single N6-methyladenosine site regulates lncRNA HOTAIR function in breast cancer cells
Source: PLoS Biol. 2022 Nov 28;20(11):e3001885. doi: 10.1371/journal.pbio.3001885 (PMC9731500; doi:10.1371/journal.pbio.3001885)

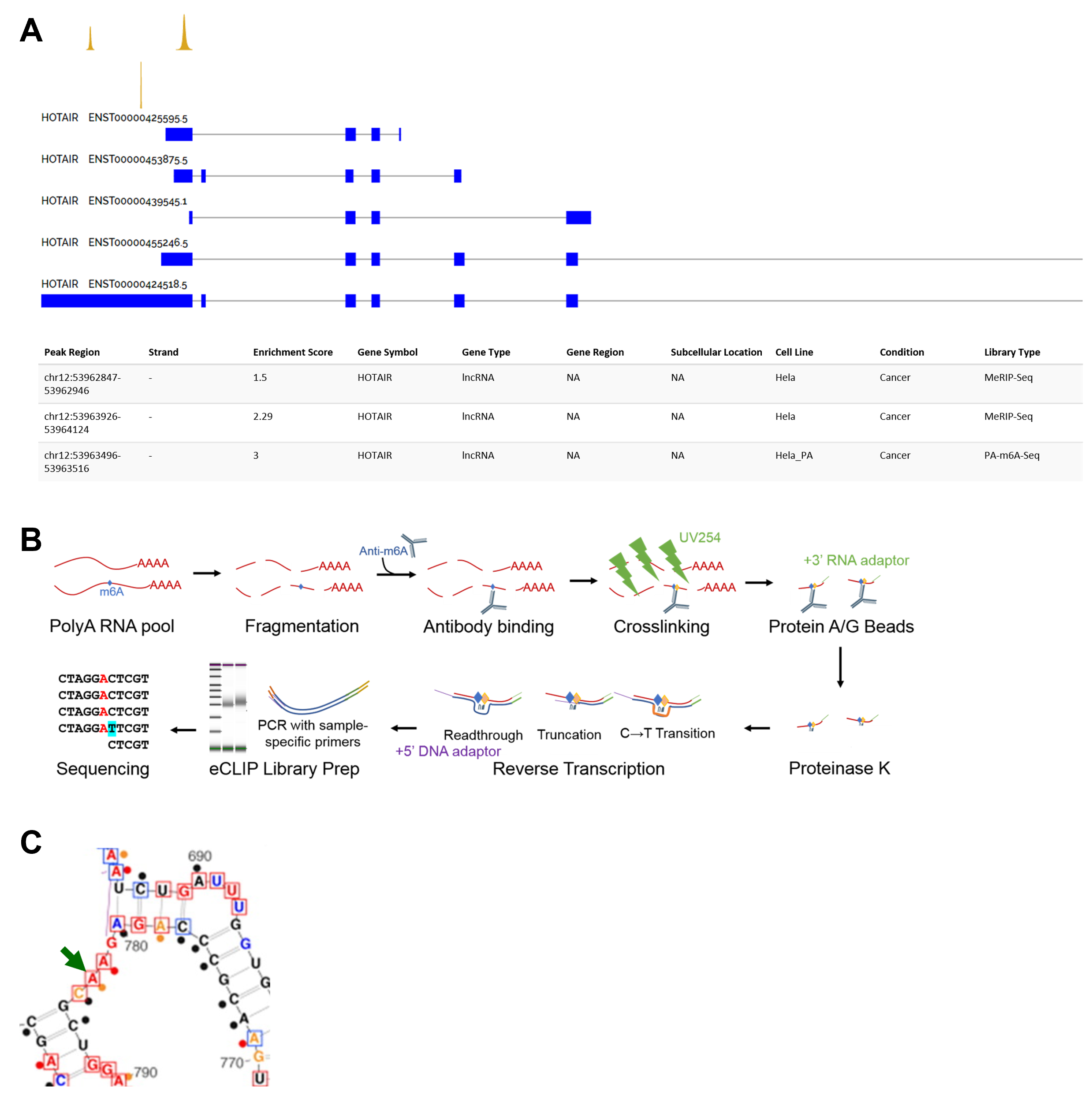

Supplement: S1 Fig — (A) CVm6A visualization of HOTAIR m6A RIP experiments. Data obtained from http://gb.whu.edu.cn:8080/CVm6A. (B) Schematic of m6A eCLIP pipeline used to map m6A sites. (C) Portion of HOTAIR structure from [16]. A783 is marked by a green arrow. (TIF) [file pbio.3001885.s004.tif]

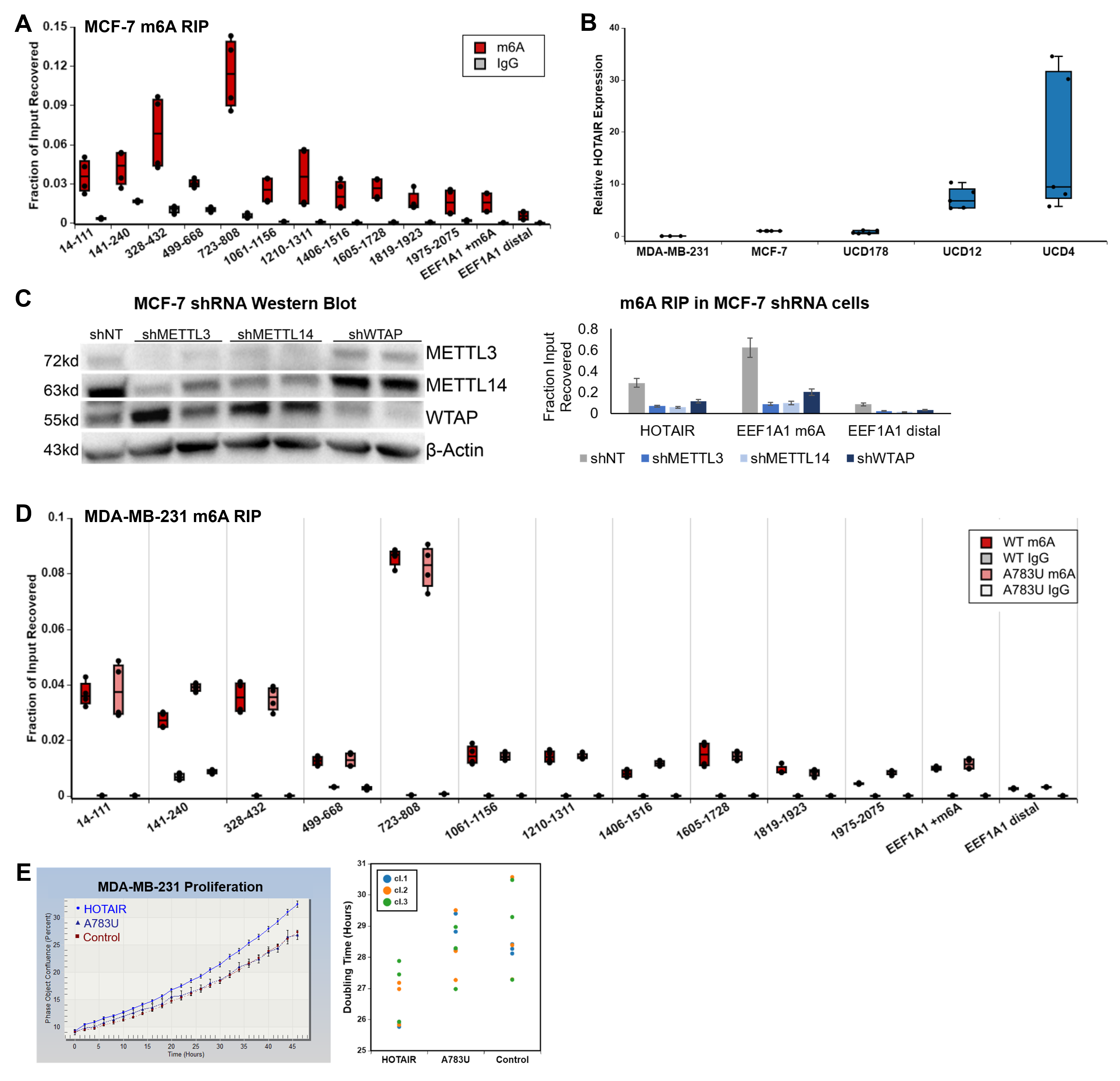

Supplement: S2 Fig — (A) m6A RNA immunoprecipitation performed with an m6A antibody or IgG control in MCF-7 breast cancer cells, quantified with probe sets spanning regions of HOTAIR and EEF1A1 as noted. Four biological replicates were performed. (B) Relative HOTAIR levels in breast cancer cell lines as determined by qRT-PCR. (C) Left, western blot of knockdown lines generated in MCF-7 cells. Right, m6A RIP results on MCF-7 knockdown lines. (D) m6A RNA immunoprecipitation performed with an m6A antibody or IgG control on MDA-MB-231 cells overexpressing WT HOTAIR, or HOTAIRA783U, quantified with probe sets spanning regions of HOTAIR and EEF1A1 as noted. Four biological replicates were performed. (E) Left, example of growth curve obtained from Incucyte experiments. Percent confluence was measured every 2 hours for 48 hours on MDA-MB-231 cell lines noted. Right, data from Fig 1F, doubling time of MDA-MB-231 overexpression cell lines, displayed as individual data points (3 clones for each cell line noted, 3 biological replicates for each clone). Numerical values in panels S2A–S2E are included in S3 Data. (TIF) [file pbio.3001885.s005.tif]

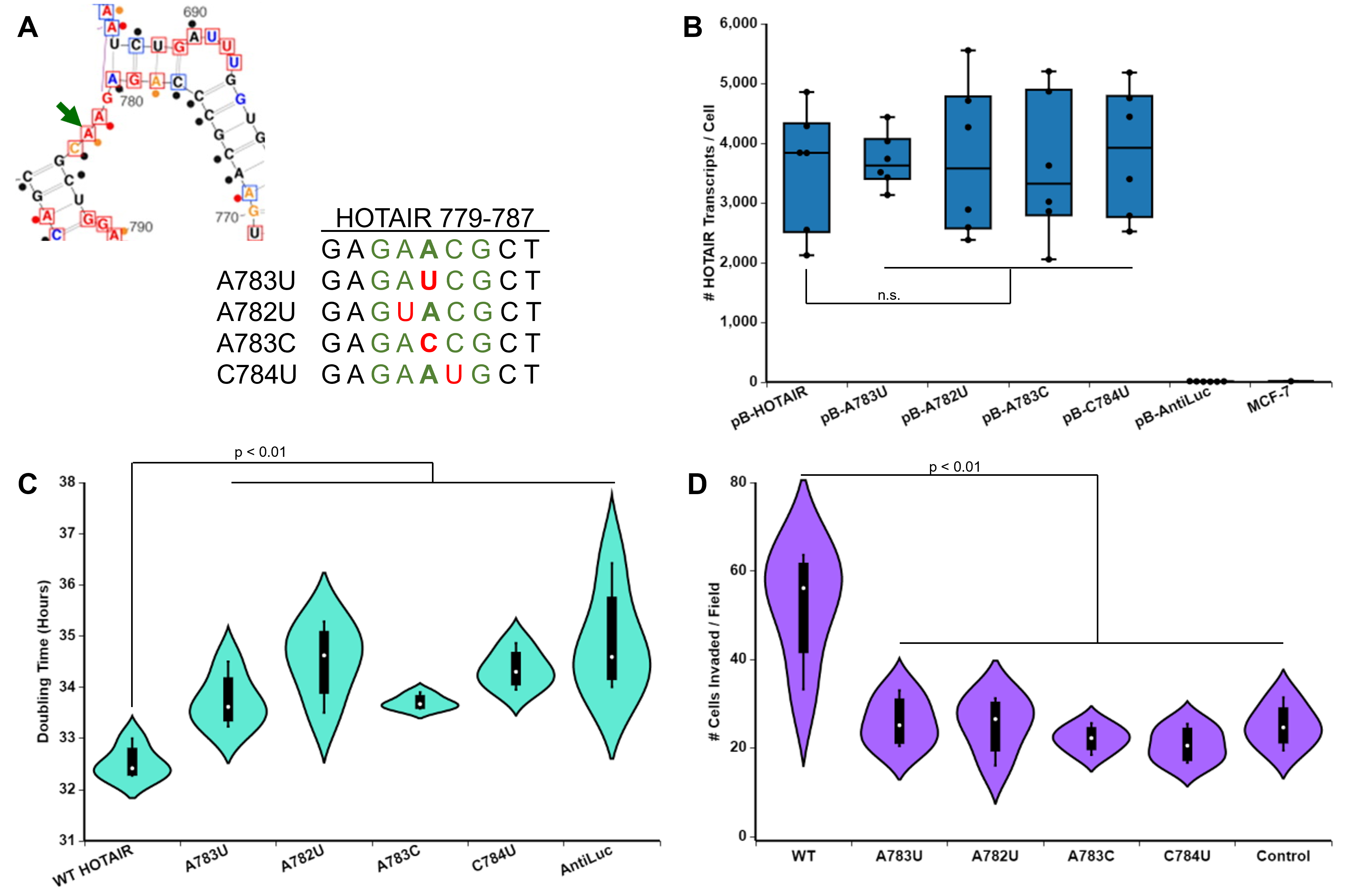

Supplement: S3 Fig — (A) Schematic of mutations to block m6A modification of A783 in HOTAIR. (B) Relative HOTAIR levels in transgenic MDA-MB-231 cell lines overexpressing WT or mutant HOTAIR or an antisense-luciferase control, or MCF-7 cells, as noted. (C) Doubling time of MDA-MB-231 overexpression cell lines noted. Experiments include 2 biological replicates each on 2 independently generated clones. (D) Quantification of Matrigel invasion assays performed with MDA-MB-231 overexpression cell lines noted. Two biological replicates each on 2 independently generated clones were performed. (E) Analysis of experiments in B–D. Numerical values in panels S3B–S3D are included in S3 Data. (TIF) [file pbio.3001885.s006.tif]

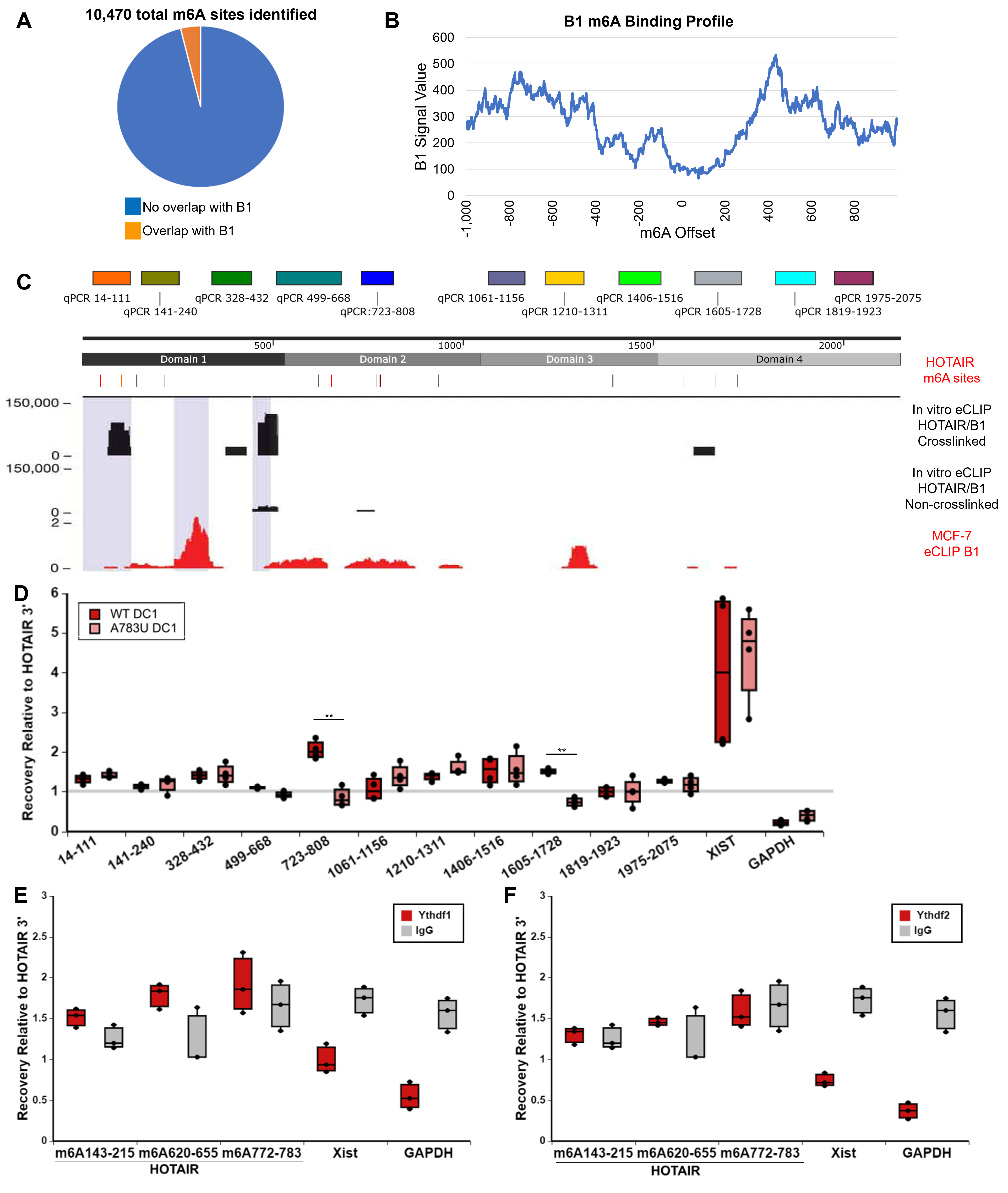

Supplement: S4 Fig — (A) Results of a search of 2,000 base pair regions surrounding m6A sites for hnRNP B1 eCLIP peaks using set functions. (B) hnRNP B1 eCLIP intensity relative to m6A sites that contain overlap with hnRNP B1 binding sites within 1,000 nucleotides upstream or downstream. (C) Map of HOTAIR qPCR probes, m6A sites, in vitro eCLIP peaks of hnRNP B1 binding, and hnRNP B1 eCLIP peaks in MCF-7 cells [13]. (D) YTHDC1 RIP performed in MDA-MB-231 cells overexpressing WT HOTAIR or HOTAIRA783U, quantified with probe sets spanning regions of HOTAIR and EEF1A1 as noted. Recovery was normalized to region 1819–1923 where no m6A sites were detected. Four biological replicates were performed. (E, F) RIP performed in MCF-7 cells with antibodies against YTHDF1 (E) or YTHDF2 (F) on 3 biological replicates. RNA recovery was monitored with qPCR probes noted in graph and divided by recovery observed with qPCR probes targeting a region of HOTAIR with no m6A (1819–1923). Numerical values in panels S4D–S4F are included in S3 Data. (TIF) [file pbio.3001885.s007.tif]

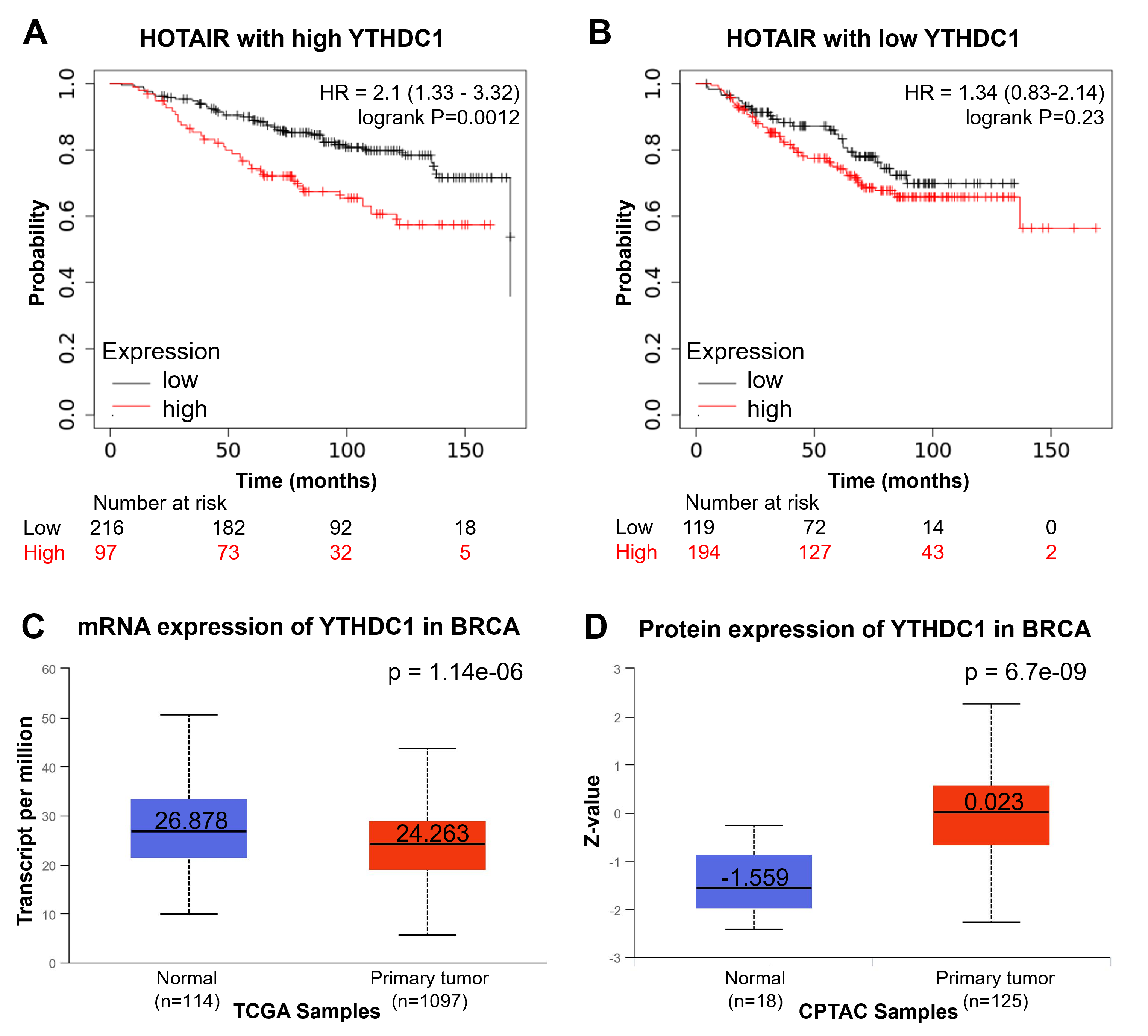

Supplement: S5 Fig — (A, B) Overall survival curves for breast cancer patients examining effect of HOTAIR on the background of either (E) high or (F) low median YTHDC1 levels, generated with Kaplan–Meier plotter [39]. (C, D) Expression of YTHDC1 (A) mRNA and (B) protein in normal breast tissue versus breast cancers generated with UALCAN [40]. (TIF) [file pbio.3001885.s008.tif]

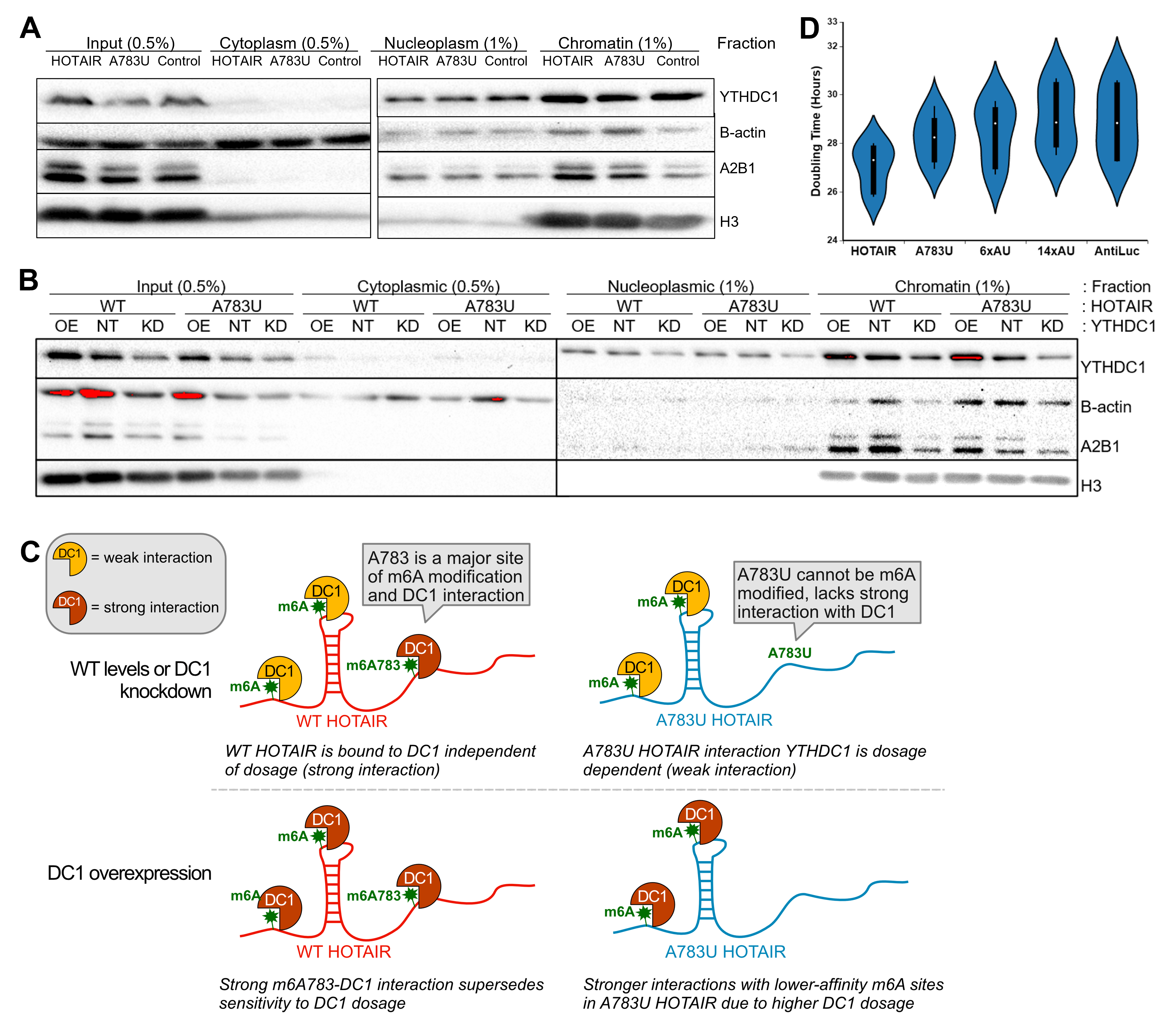

Supplement: S6 Fig — (A) Western blot on fractionation of MDA-MB-231 cell lines overexpressing WT or A783U HOTAIR or antisense-luciferase. (B) Western blot performed on fractionation of MDA-MB-231 cell lines overexpressing WT or A783U HOTAIR containing overexpression (OE), non-targeting (NT), or knock-down (KD) of YTHDC1. (C) Model for differences observed between WT and A783U HOTAIR upon knockdown and overexpression of YTHDC1. (D) Doubling time of MDA-MB-231 cells expressing WT, A783U, 6xAU, or 14xAU HOTAIR, or an anti-luciferase control. Three biological replicates each were performed on 2 independently generated clones. Numerical values in panels S6D are included in S3 Data. (TIF) [file pbio.3001885.s009.tif]

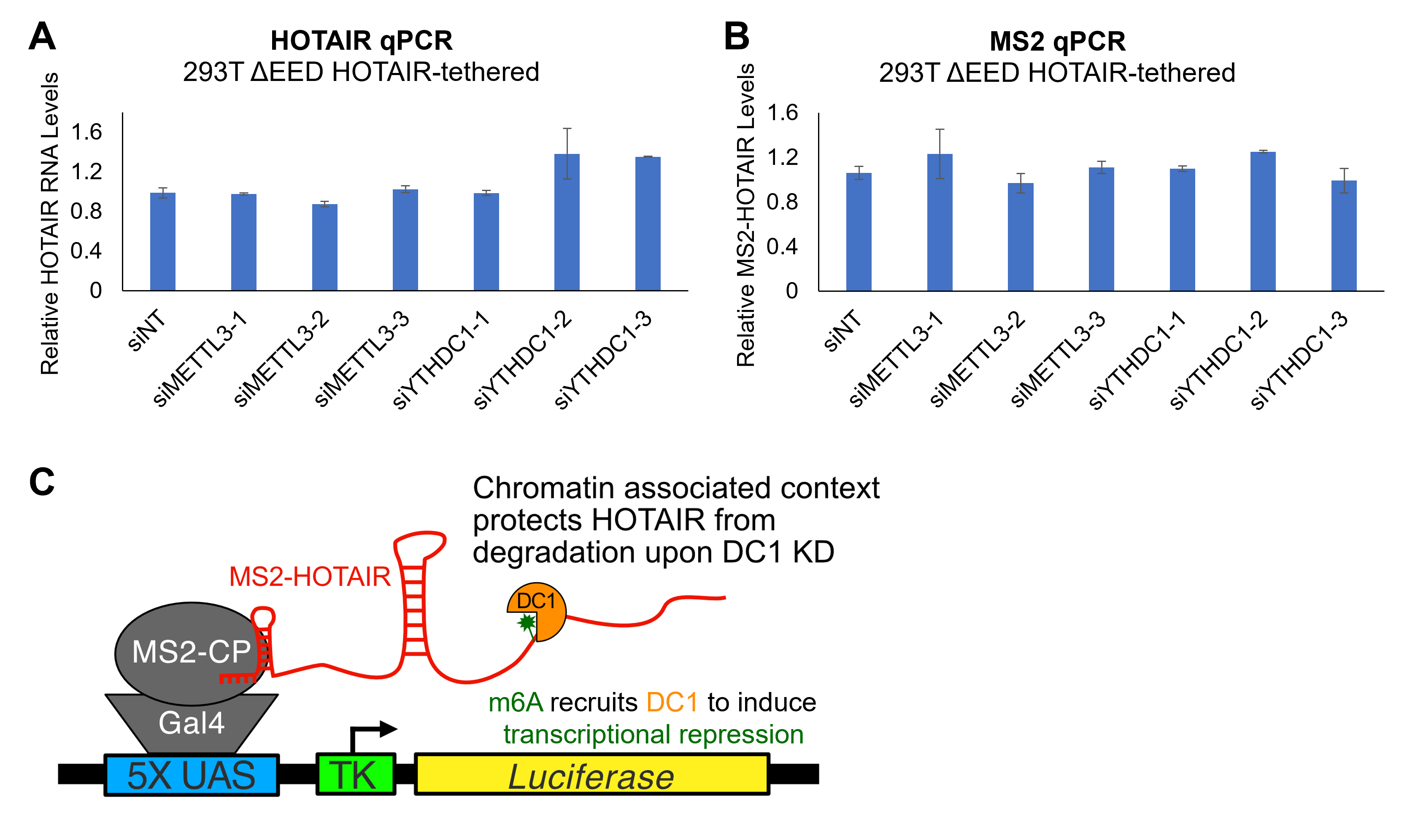

Supplement: S7 Fig — (A, B) qRT-PCR of HOTAIR (A) or MS2 (B) in 293T HOTAIR-tethered cells lacking EED with siRNA knockdown of METTL3 or YTHDC1 or a non-targeting control. (C) Model for HOTAIR stability in chromatin-tethered context. Numerical values in panels S7A–S7B are included in S3 Data. (TIF) [file pbio.3001885.s010.tif]

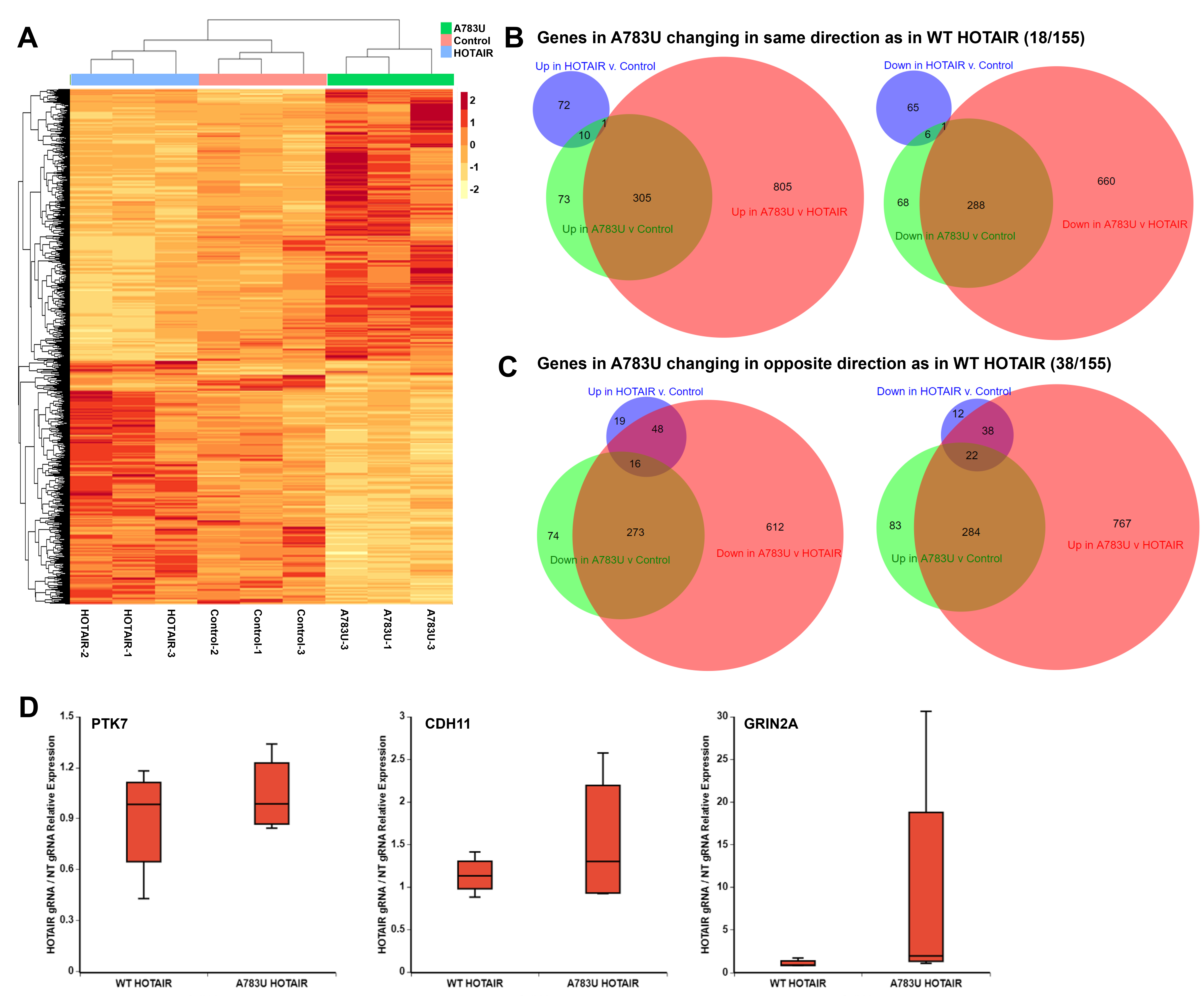

Supplement: S8 Fig — (A) Heat map of Z-scores of all DEGs identified in pairwise comparisons. (B, C) Venn diagrams (created using BioVenn [66]) of comparisons of DEGs by expression pattern noted. (D) Relative expression of HOTAIR gRNA samples compared to NT gRNA samples for WT HOTAIR-up-regulated genes noted. Numerical values in panel S8D are included in S3 Data. (TIF) [file pbio.3001885.s011.tif]
