# Supplementary material for: A single N6-methyladenosine site regulates lncRNA HOTAIR function in breast cancer cells
Source: PLoS Biol. 2022 Nov 28;20(11):e3001885. doi: 10.1371/journal.pbio.3001885 (PMC9731500; doi:10.1371/journal.pbio.3001885)
Supplement: S1 Table — Each column represents a single experiment with the cell line noted. Each row is an m6A site detected within HOTAIR and includes Nt # (location within HOTAIR transcript) and chromosome position. Each X represents an m6A site detected in HOTAIR in each experiment. Using thresholding from [31], “X” represents high confidence m6A sites (≥3 C➔T mutations following the m6A site in ≥5% of reads), “x” represents low confidence sites (≥3 C➔T mutations following the m6A site in ≥2.5% of reads), and “*” represents sites called with reduced threshold of at least 2 C➔T mutation events detected. HOTAIR m6A site positions in bold were included in the 6× HOTAIR mutant, while the remainder of sites (not including Nt 557) were included in the 14× HOTAIR mutant. Note that MCF-7 replicate 3 was a lower-depth run used as a point of comparison to the higher-depth replicates 1 and 2 in [31]. HOTAIR read depth in MCF-7 experiments is approximately 10× less than when overexpressed in MDA-MB-231 cells. m6A site 783 is highlighted in red. (DOCX) [file pbio.3001885.s012.docx]

**Table S1**

|  | Experiments | | | | | | | | | | |
| --- | --- | --- | --- | --- | --- | --- | --- | --- | --- | --- | --- |
| HOTAIR m6A site positions | MCF-7 rep1 | MCF-7 rep2 | MCF-7 rep3 | MDA-MB-231 pB-HOTAIR rep1 | MDA-MB-231 pB-HOTAIR rep2 | MDA-MB-231 pB-HOTAIR rep3 | MDA-MB-231 pB-HOTAIR^A783U^ rep1 | MDA-MB-231 pB-HOTAIR^A783U^ rep2 | MDA-MB-231 pB-HOTAIR^A783U^ rep3 | MDA-MB-231 pB-Anti-Luc | 293 HOTAIR-Luc dEED |
| **Nt 48 /**  **54362413** |  |  |  | **X** | **X** |  | **X** | x | **X** | **X** | x |
| **Nt 102 / 54361137** |  |  |  | **X** | x |  | x | ***** | ***** |  | x |
| Nt 143 / 54361096 |  |  |  | * | * |  |  |  |  |  | x |
| Nt 215 / 54360133 |  |  |  | * |  |  | x |  |  |  | x |
| Nt 557 / 54357821 |  |  |  | * |  |  |  | x |  |  |  |
| Nt 620 / 54357758 |  |  |  | **X** | x | * |  | x |  |  | * |
| **Nt 655 / 54357723** |  |  |  | **X** | **X** | **X** | **X** |  | ***** | **X** | x |
| **Nt 772 / 54357606** |  |  |  | x | ***** | x | x | **X** | x | **X** | x |
| **Nt 783 / 54357595** | **X** | ***** |  | **X** | **X** | **X** |  |  |  | **X** | **X** |
| Nt 936 / 54357442 |  |  |  | * |  |  | x |  |  |  |  |
| Nt 1394 / 54356983 |  |  |  | x |  |  | * |  |  |  | x |
| Nt 1579 / 54356798 |  |  |  | **X** |  |  |  |  |  |  | * |
| Nt 1663 / 54356714 |  |  |  | x |  |  |  |  |  | ***** |  |
| Nt 1722 / 54356655 |  |  |  | ***** |  |  |  | ***** |  |  | x |
| **Nt 1739 / 54356638** |  |  |  | x | **X** |  | **X** | ***** |  |  | x |

Legend: **X**=high confidence x=low confidence *=reduced threshold
