# Supplementary material for: A single N6-methyladenosine site regulates lncRNA HOTAIR function in breast cancer cells
Source: PLoS Biol. 2022 Nov 28;20(11):e3001885. doi: 10.1371/journal.pbio.3001885 (PMC9731500; doi:10.1371/journal.pbio.3001885)
Supplement: S2 Table — X indicates an m6A site detected in 2+ replicates in the cell line noted. (DOCX) [file pbio.3001885.s013.docx]

**Table S2**

| HOTAIR m6A sites | Sequence | MCF-7 | MDA-MB-231 pB-HOTAIR | MDA-MB-231 pB-HOTAIR^A783U^ |
| --- | --- | --- | --- | --- |
| **Nt 48 / 54362413** | GGACU |  | X | X |
| **Nt 102 / 54361137** | AGACC |  | X | X |
| Nt 143 / 54361096 | AGACC |  | X |  |
| Nt 620 / 54357758 | GAACA |  | X |  |
| **Nt 655 / 54357723** | AGACA |  | X | X |
| **Nt 772 / 54357606** | GAACG |  | X | X |
| **Nt 783 / 54357595** | GAACG | X | X |  |
| **Nt 1739 / 54356638** | GGACU |  | X | X |
