# Supplementary material for: A single N6-methyladenosine site regulates lncRNA HOTAIR function in breast cancer cells
Source: PLoS Biol. 2022 Nov 28;20(11):e3001885. doi: 10.1371/journal.pbio.3001885 (PMC9731500; doi:10.1371/journal.pbio.3001885)
Supplement: S3 Table — Each meCLIP experiment is listed with the UCD cell line, detection of m6A783, number of raw reads over HOTAIR A783, and number of C➔T conversions following A783. The last column lists the transcripts per million and raw reads obtained in RNA seq experiments in [33]. (DOCX) [file pbio.3001885.s014.docx]

**Table S3**

| **Cell Line** | **m6A783** | **Raw Reads (Conversions)** | **Transcripts per million (reads)^33^** |
| --- | --- | --- | --- |
| **UCD4** | Yes | 66 (6) | 6.7 (85) |
| **UCD12 (REP1)** | No | 38 (0) | 2.9 (48) |
| **UCD12 (REP2)** | No | 33 (1) | 2.9 (48) |
| **UCD178 (REP1)** | No | 16 (0) | 0.4 (8) |
| **UCD178 (REP2)** | No | 16 (0) | 0.4 (8) |
