# Supplementary material for: A single N6-methyladenosine site regulates lncRNA HOTAIR function in breast cancer cells
Source: PLoS Biol. 2022 Nov 28;20(11):e3001885. doi: 10.1371/journal.pbio.3001885 (PMC9731500; doi:10.1371/journal.pbio.3001885)
Supplement: S4 Table — (DOCX) [file pbio.3001885.s015.docx]

**Table S4**

| **Gene / Knockdown or Overexpression** | **shRNA/ORF Used** |
| --- | --- |
| METTL3 Knockdown | TRCN0000034715, TRCN0000034717 |
| METTL14 Knockdown | TRCN0000015933, TRCN0000015937 |
| WTAP Knockdown | TRCN0000231422, TRCN0000231424 |
| YTHDC1 Knockdown | TRCN0000243987, TRCN0000243989 |
| YTHDC1 Overexpression | ORF clone ccsdBroad304_04559 |
