# Supplementary material for: A single N6-methyladenosine site regulates lncRNA HOTAIR function in breast cancer cells
Source: PLoS Biol. 2022 Nov 28;20(11):e3001885. doi: 10.1371/journal.pbio.3001885 (PMC9731500; doi:10.1371/journal.pbio.3001885)
Supplement: S5 Table — (DOCX) [file pbio.3001885.s016.docx]

**Table S5**

| **Experiment** | **Forward Oligo** | **Reverse Oligo** |
| --- | --- | --- |
| A783U HOTAIR QuikChange | AG66  CGCCCAGAGAtCGCTGGAAAAACCTGAGCGG | AG67  CCAGCGaTCTCTGGGCGTTCATGTGGCGAGC |
| A783U HOTAIR pBABE-Puro | AG68  CCTAAACCAGCAATTACACCCAAGCTCGTTGGGGCCTAAG | AG69  CTGTGCTGGCGAATTCCTACGTACCACCACACTGGGATCCGAAAATGCATCCAGATATTAAT |
| A782U HOTAIR QuikChange | AG148  CGCCCAGAGtACGCTGGAAAAACCTGAGCGG | AG149  CCAGCGTACTCTGGGCGTTCATGTGGCGAGC |
| A783C HOTAIR QuikChange | AG150  CGCCCAGAGACCGCTGGAAAAACCTGAGCGG | AG151  CCAGCGGTCTCTGGGCGTTCATGTGGCGAGC |
| C784U HOTAIR QuikChange | AG154  CGCCCAGAGAATGCTGGAAAAACCTGAGCGG | AG155  CCAGCaTTCTCTGGGCGTTCATGTGGCGAGC |
| Cloning YTHDC1 into pCDNA3-FLAG | AG64  ggtaccGCGGCTGACAGTCGGGAGGAG | AG65  gcggccgccCTAATCTTCTATATCGACCTCTC |
| NheI cloning YTHDC1 into pXR002 | AL01  gtagctgctagcGACTACAAAGACGATGACGATAAAGGGG | AL02  gatgctgctagcTCTTCTATATCGACCTCTCTCCCCTCG |
| HOTAIR gRNA cloning into pXR003 | AL05  AAACCCCCGGCACCCGCTCAGGTTTT | AL10  AAAAAAAACCTGAGCGGGTGCCGGGG |
| Non-targeting gRNA cloning into pXR003 | AL07  AAACCAGAAGCGTACCATACTCACGA | AL 11  AAAATCGTGAGTATGGTACGCTTCTG |
