# Supplementary material for: A single N6-methyladenosine site regulates lncRNA HOTAIR function in breast cancer cells
Source: PLoS Biol. 2022 Nov 28;20(11):e3001885. doi: 10.1371/journal.pbio.3001885 (PMC9731500; doi:10.1371/journal.pbio.3001885)
Supplement: S6 Table — (DOCX) [file pbio.3001885.s017.docx]

**Table S6**

| **m6A eCLIP oligonucleotides** | **Sequence** |
| --- | --- |
| X1a (RNA) | /5Phos/rArUrArUrArGrG rNrNrNrNrN rArGrArUrCrGrGrArArGrArGrCrGrUrCrGrUrGrUrArG/3SpC3/ |
| X1b (RNA) | /5Phos/rArArUrArGrCrA rNrNrNrNrN rArGrArUrCrGrGrArArGrArGrCrGrUrCrGrUrGrUrArG/3SpC3/ |
| Rand3Tr3 (RNA) | /5phos/rArGrArUrCrGrGrArArGrArGrCrGrUrCrGrUrG/3SpC3/ |
| RiL19 (RNA) | /5phos/rArGrArUrCrGrGrArArGrArGrCrGrUrCrGrUrG/3SpC3/ |
| AR17 (DNA) | /5Phos/NNNNNNNNNNAGATCGGAAGAGCACACGTCTG/3SpC3/ |
