# Supplementary material for: A single N6-methyladenosine site regulates lncRNA HOTAIR function in breast cancer cells
Source: PLoS Biol. 2022 Nov 28;20(11):e3001885. doi: 10.1371/journal.pbio.3001885 (PMC9731500; doi:10.1371/journal.pbio.3001885)
Supplement: S7 Table — (DOCX) [file pbio.3001885.s018.docx]

**Table S7**

| **Gene** | **qPCR Forward Primer** | **qPCR Reverse Primer** |
| --- | --- | --- |
| HOTAIR (14-111) | TCTGGAGCTTGATCCGAAAG | GGTGTTGGTCTGTGGAACT |
| HOTAIR (141-240)^9^ | AGACCCTCAGGTCCCTAATATC | CCCTACTGCAGGCTTCTAAATC |
| HOTAIR (328-432) | GTCTGATGTTTACAAGACCAGAAATG | CTCTCGCCAATGTGCATACTTA |
| HOTAIR (499-668)^15^ | GGTAGAAAAAGCAACCACGAAGC | ACATAAACCTCTGTCTGTGAGTGCC |
| HOTAIR (723-808) | GGGAACGGGAGTACAGAGAGAATA | GGCACCCGCTCAGGTTT |
| HOTAIR (1061-1156) | CGGAACCCATGGACTCATAAA | TCCAGAACCCTCTGACATTTG |
| HOTAIR (1210-1311) | GAGTCCGTTCAGTGTCAGAAA | ACACAAGTAGCAGGGAAAGG |
| HOTAIR (1406-1516) | CCGGAATTTGAGAGGAACATAGA | AAAGGCTAGGGCTGGTTTC |
| HOTAIR (1605-1728) | GCAGCACAGAGCAACTCTATAA | CAGGGTCCCACTGCATAATC |
| HOTAIR (1819-1923) | TGATGCATGTAGACACAGAAGG | TCAGGCATTGGGAATGGTAAT |
| HOTAIR (1975-2075) | GAACCCAGAAGAACGCAATTT | TGCATACCTACCCAATGTATGG |
| GAPDH | CCGGGAAACTGTGGCGTGATGG | AGGTGGAGGAGTGGGTGTCGCTGTT |
| XIST | AAACCCAACACGAAAAGCAC | GCGGTCACACAGGAAAAGAT |
| EEF1A1 +m6A | CGGTCTCAGAACTGTTTGTTTC | AAACCAAAGTGGTCCACAAA |
| EEF1A1 distal | GGATGGAAAGTCACCCGTAAG | TTGTCAGTTGGACGAGTTGG |
| PTK7 | ACACTTCGTTGCCACATTGAT | CAGCAGGAATACAGCCCAC |
| CDH11 | AGAGGTCCAATGTGGGAACG | GGTTGTCCTTCGAGGATACTGT |
| GRIN2A | TGGCCTCACCGGGTATGATT | CAATGCCGTCCCTCACTCTC |
| SEMA5A | GATCCTGCCATTTACCGAAGC | AGATGACACAAAGTTTGGCTCA |
| SIRPA | ACATGGTCCACCTCAACCG | ACGCTGGCGTACTCTGAGA |
| TP53I11 | GAAGACCCGCAAGATCCTCG | TTTCATTGCCTAAGACCTGGC |
| Luciferase (LucR2) | GCACTGATCATGAACTCCTCTGGATCTAC | GAGAATAGGGTTGGCACCAGCAGCGCAC |
