# Supplementary material for: A single N6-methyladenosine site regulates lncRNA HOTAIR function in breast cancer cells
Source: PLoS Biol. 2022 Nov 28;20(11):e3001885. doi: 10.1371/journal.pbio.3001885 (PMC9731500; doi:10.1371/journal.pbio.3001885)
Supplement: S8 Table — (DOCX) [file pbio.3001885.s019.docx]

**Table S8**

| **Fragment** | **Template** | **F Primer** | **R Primer** |
| --- | --- | --- | --- |
| WT HOTAIR D2 | pAJ249 | MB88  GTAATACGACTCACTATAGGGAGCCAGAGGAG | MB89  CCATATAAACTCCTTAAAGCTTATATTTTACAGTCC |
| A783U HOTAIR D2 | pAJ385 |  |  |
| RAT-WT D2 | WT HOTAIR D2 | MB22  TAATACGACTCACTATAGGG | MB94  CGATGGCACGAGTGTAGCTAAACCTCGTGCCGACGTCTAAGGGTTTCCATATAAACTCCTTAAAGCTT |
| RAT-A783U D2 | A783U HOTAIR D2 |  |  |
