# Supplementary material for: A single N6-methyladenosine site regulates lncRNA HOTAIR function in breast cancer cells
Source: PLoS Biol. 2022 Nov 28;20(11):e3001885. doi: 10.1371/journal.pbio.3001885 (PMC9731500; doi:10.1371/journal.pbio.3001885)

Figure 2F anti-FLAG blot

upper band = YTHDC1-FLAG
lower band = IgG

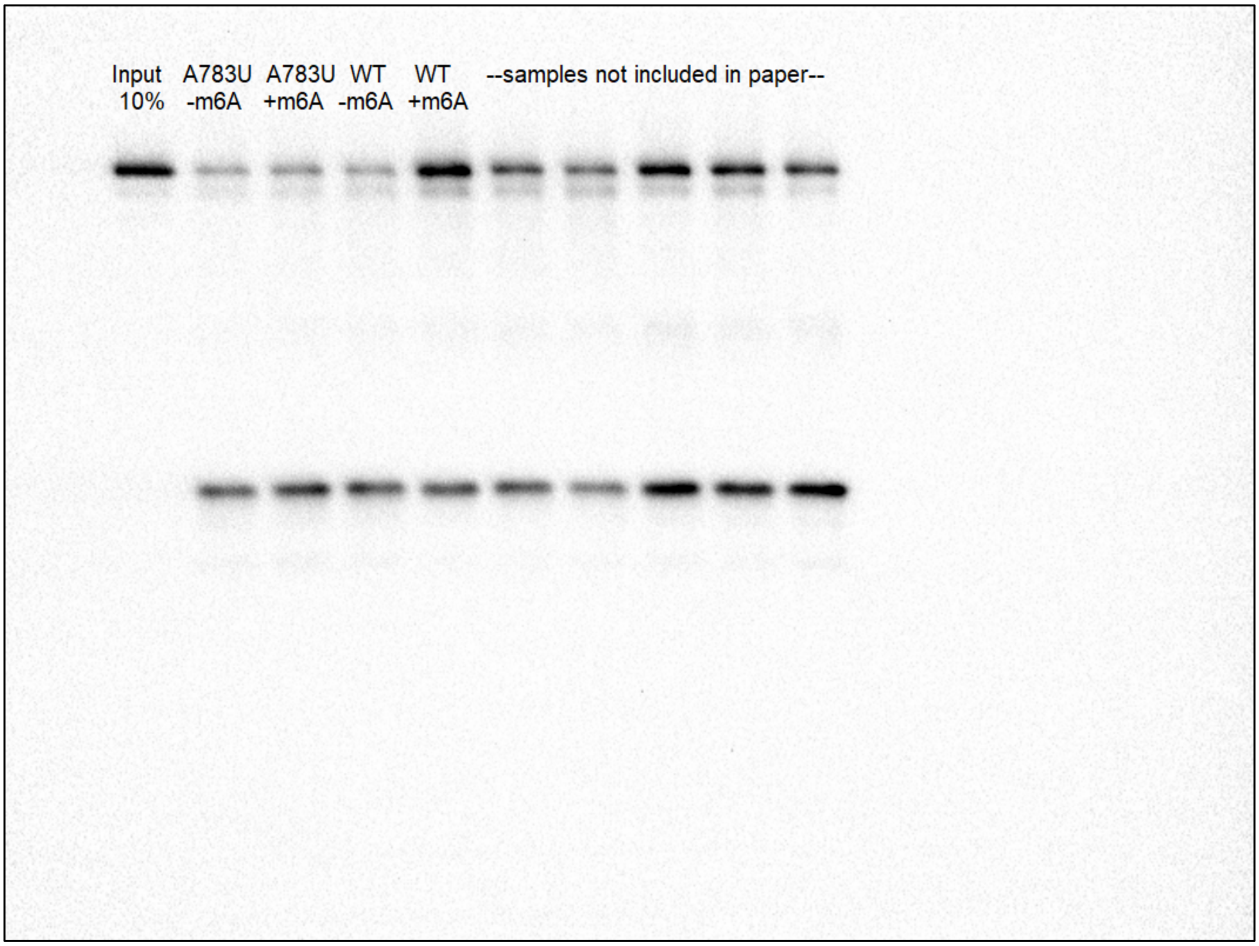

Figure 3A B-actin blot

Samples:

1. MDA-MB-231 pB-HOTAIR pLX-DC1
2. MDA-MB-231 pB-HOTAIR shNT
3. MDA-MB-231 pB-HOTAIR shDC1
4. MDA-MB-231 pB-A783U pLX-DC1
5. MDA-MB-231 pB-A783U shNT
6. MDA-MB-231 pB-A783U shNT

7-9 not included in publication

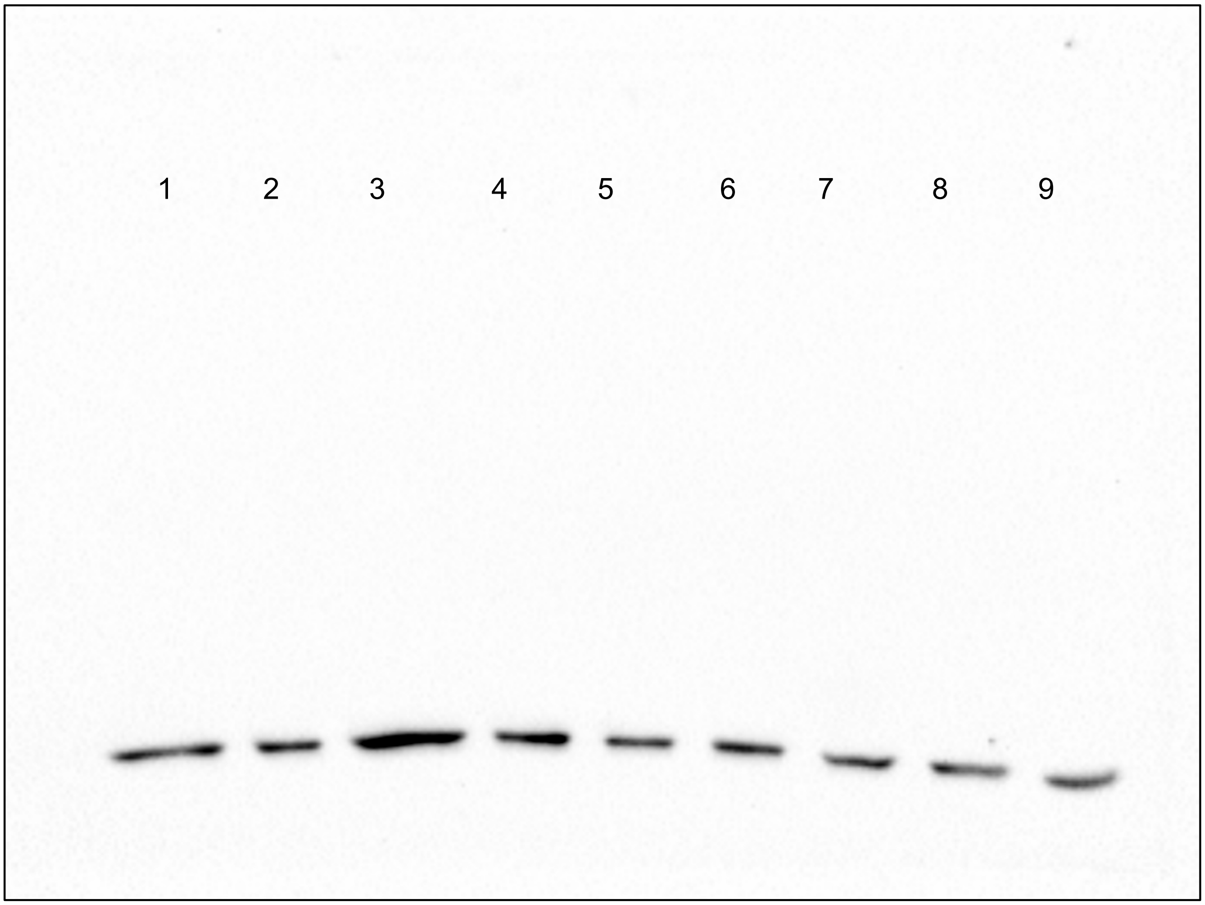

Figure 3A YTHDC1 blot

Samples:

1. MDA-MB-231 pB-HOTAIR pLX-DC1
2. MDA-MB-231 pB-HOTAIR shNT
3. MDA-MB-231 pB-HOTAIR shDC1
4. MDA-MB-231 pB-A783U pLX-DC1
5. MDA-MB-231 pB-A783U shNT
6. MDA-MB-231 pB-A783U shNT

7-9 not included in publication

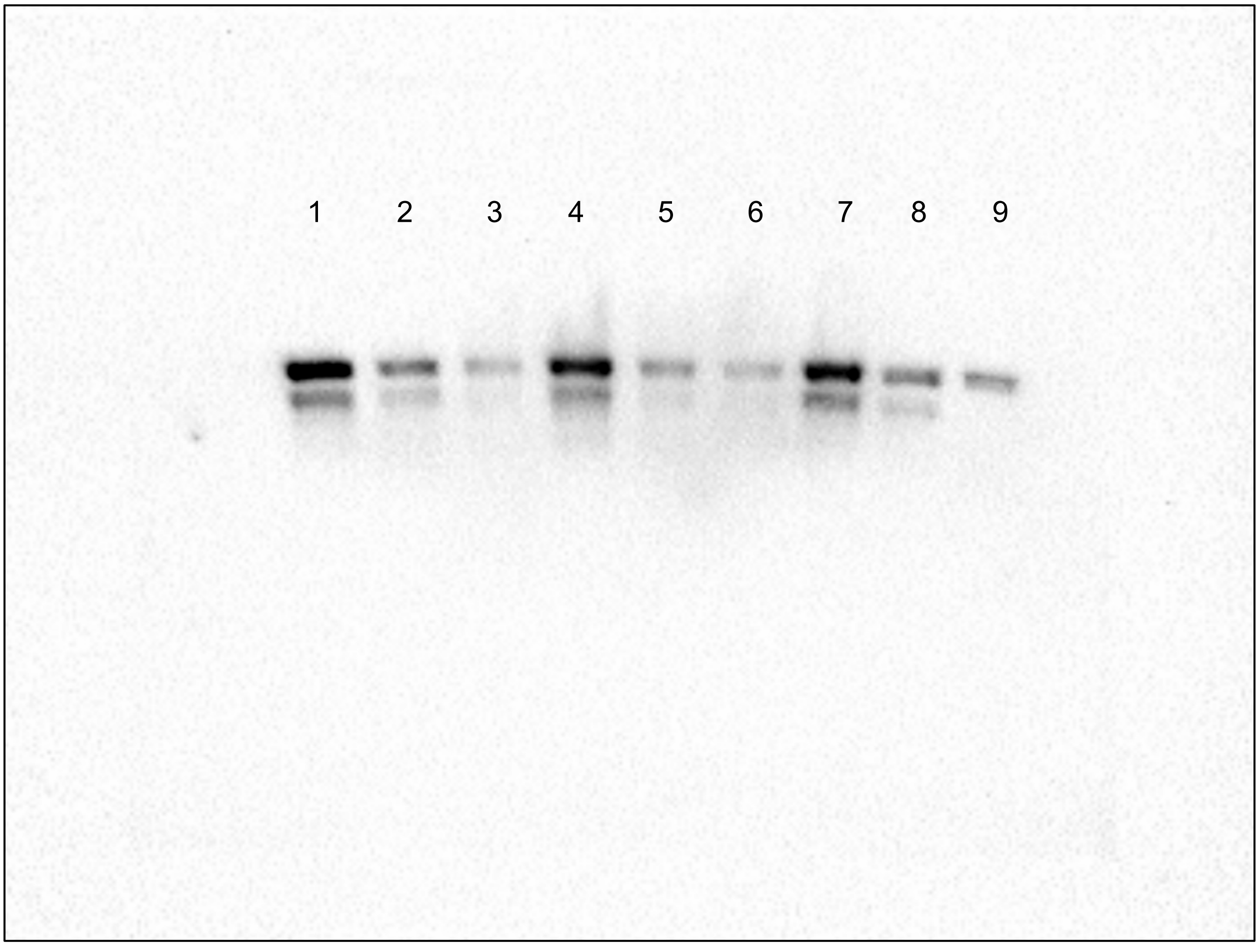

Figure 4D B-actin blot

Samples:

1. siMETTL3-1
2. siMETTL3-2
3. siMETTL3-3

8. siNT

4-7, 9 not included

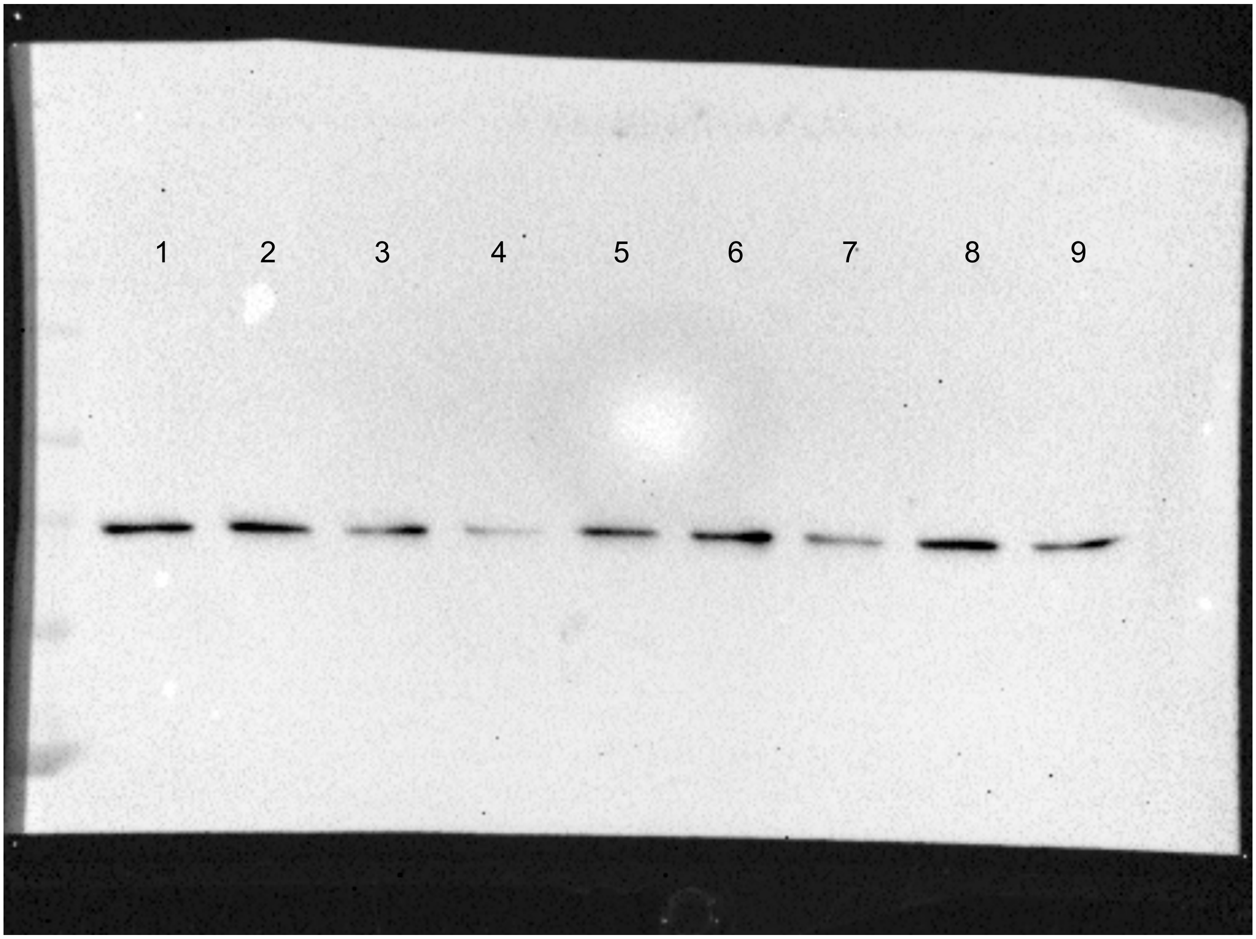

Figure 4D Mettl3 blot

Samples:

4. siMETTL3-1
5. siMETTL3-2
6. siMETTL3-3

8. siNT

4-7, 9 not included

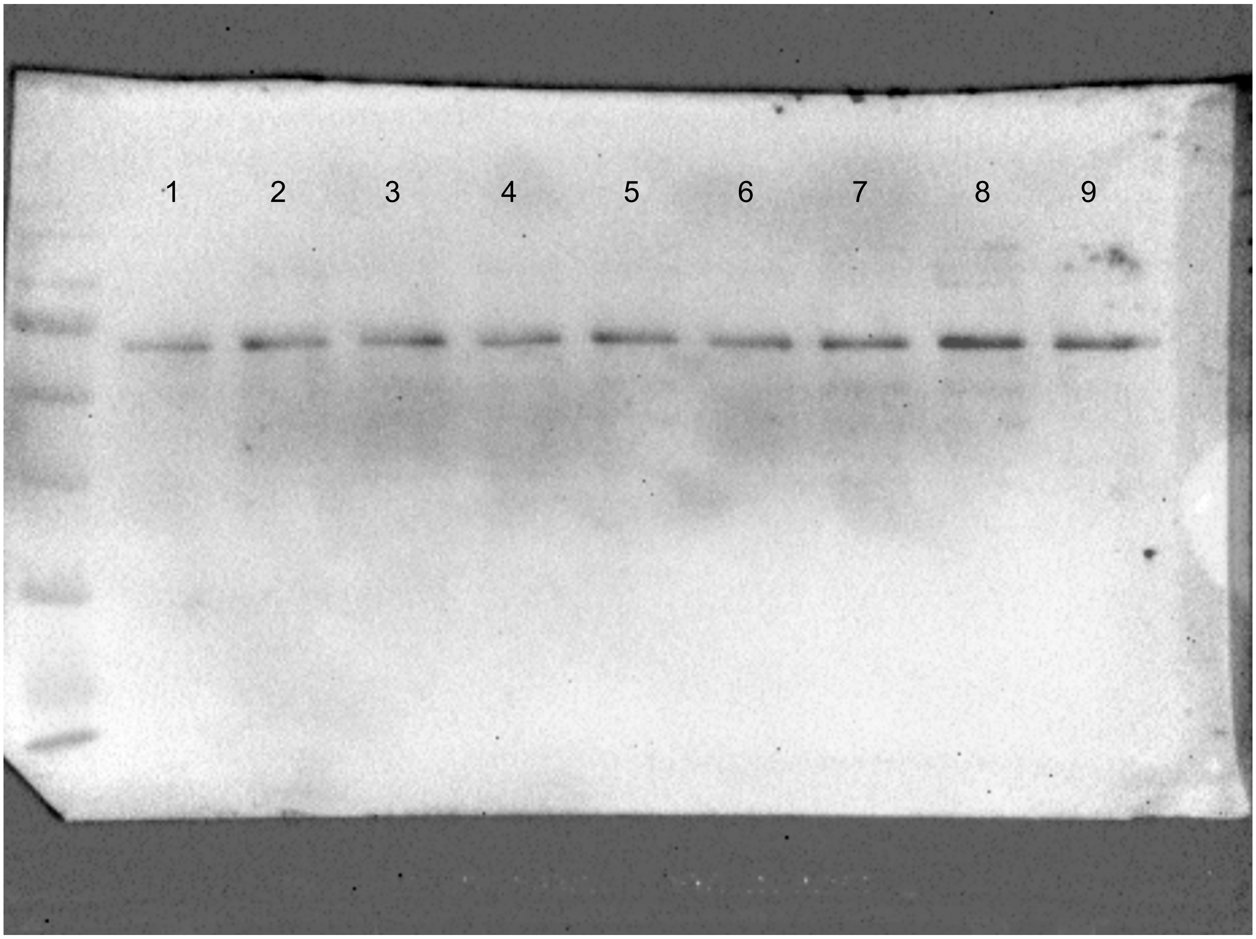

Figure 4G B-actin blot

Samples:

1. siYTHDC1-1
2. siYTHDC1-2
3. siYTHDC1-3

7. siNT

4-7, 9 not included

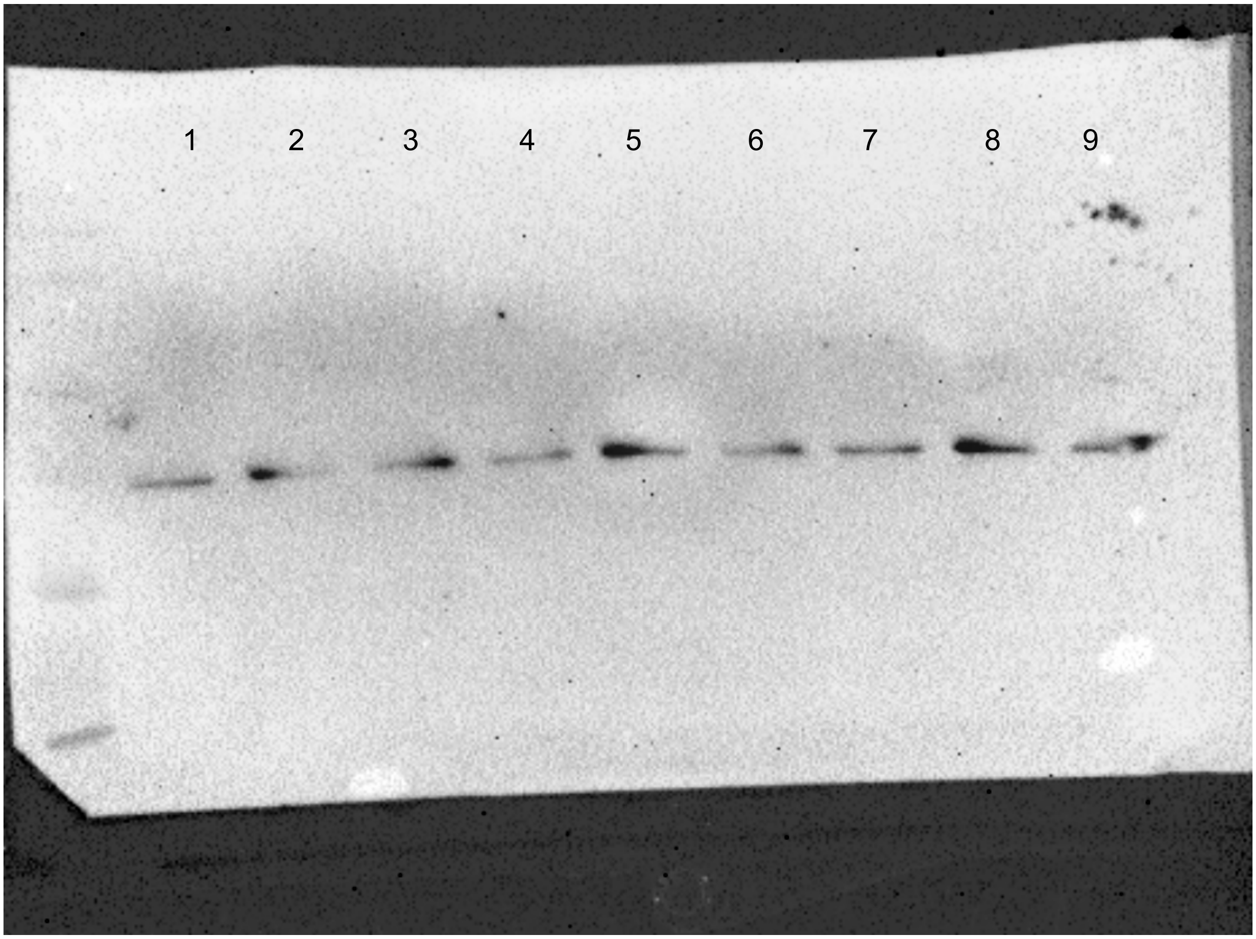

Figure 4G YTHDC1 blot

Samples:

1. siYTHDC1-1
2. siYTHDC1-2
3. siYTHDC1-3

7. siNT

4-7, 9 not included

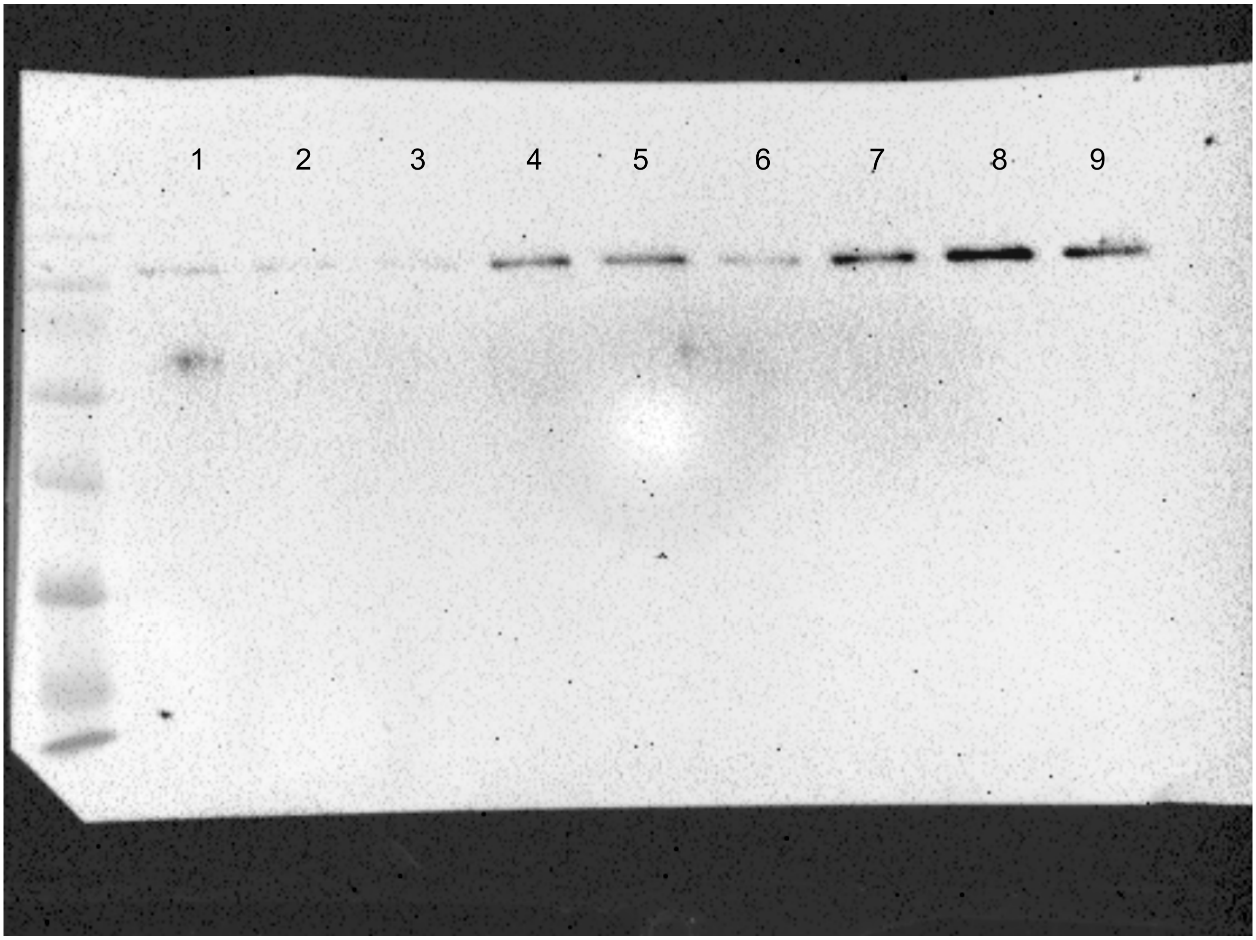

Figure 6B B-actin blot

Samples:

1. MDA-MB-231 pB-HOTAIR (WT) + dCasRX-DC1 + HOTAIR gRNA
2. MDA-MB-231 pB-HOTAIR (A783U) + dCasRX-DC1 + HOTAIR gRNA
3. MDA-MB-231 pB-HOTAIR (WT) + dCasRX-DC1 + NT gRNA
4. MDA-MB-231 pB-HOTAIR (A783U) + dCasRX-DC1 + NT gRNA

5-12 not included in publication

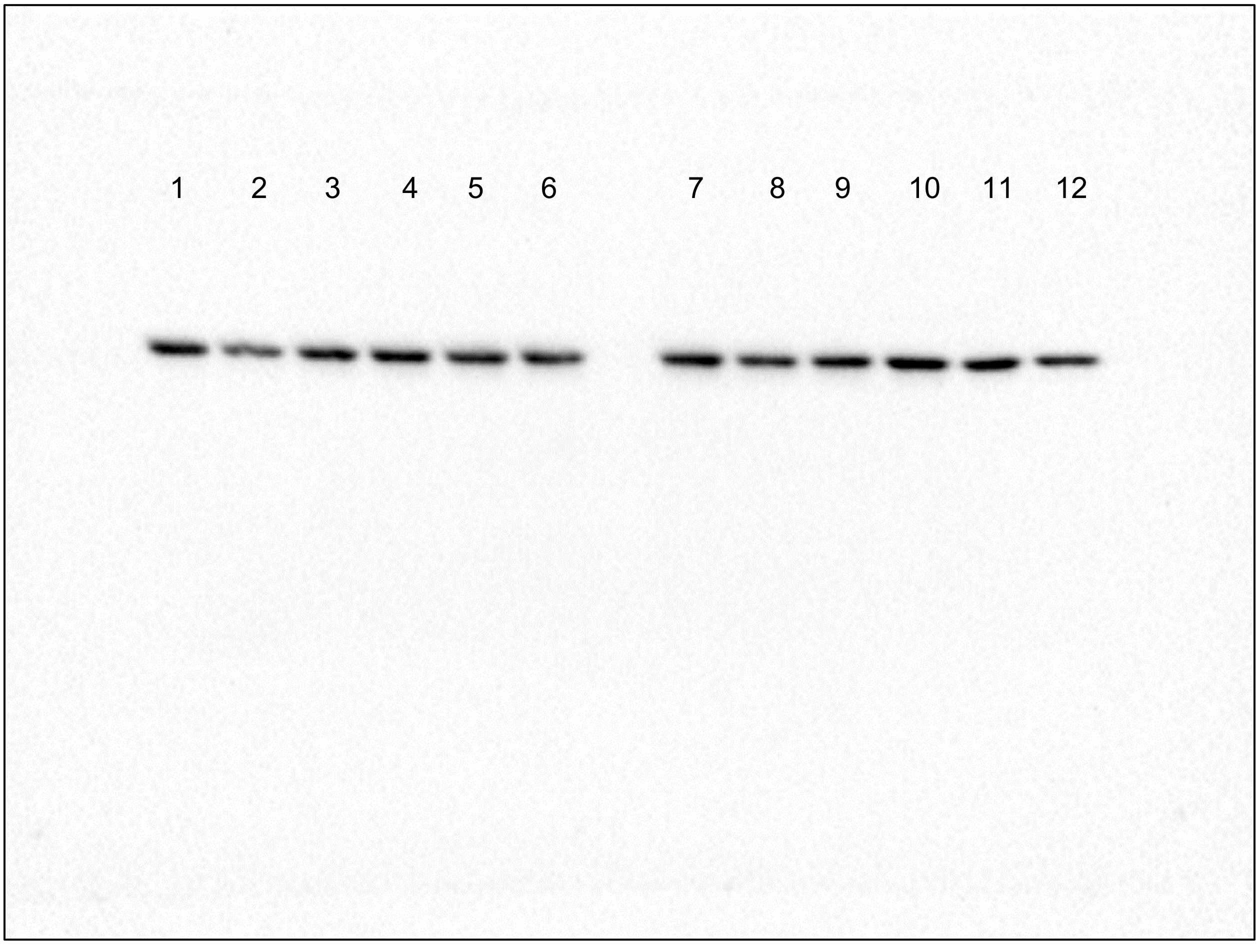

Figure 6B YTHDC1 blot

Samples:

1. MDA-MB-231 pB-HOTAIR (WT) + dCasRX-DC1 + HOTAIR gRNA
2. MDA-MB-231 pB-HOTAIR (A783U) + dCasRX-DC1 + HOTAIR gRNA
3. MDA-MB-231 pB-HOTAIR (WT) + dCasRX-DC1 + NT gRNA
4. MDA-MB-231 pB-HOTAIR (A783U) + dCasRX-DC1 + NT gRNA

5-12 not included in publication

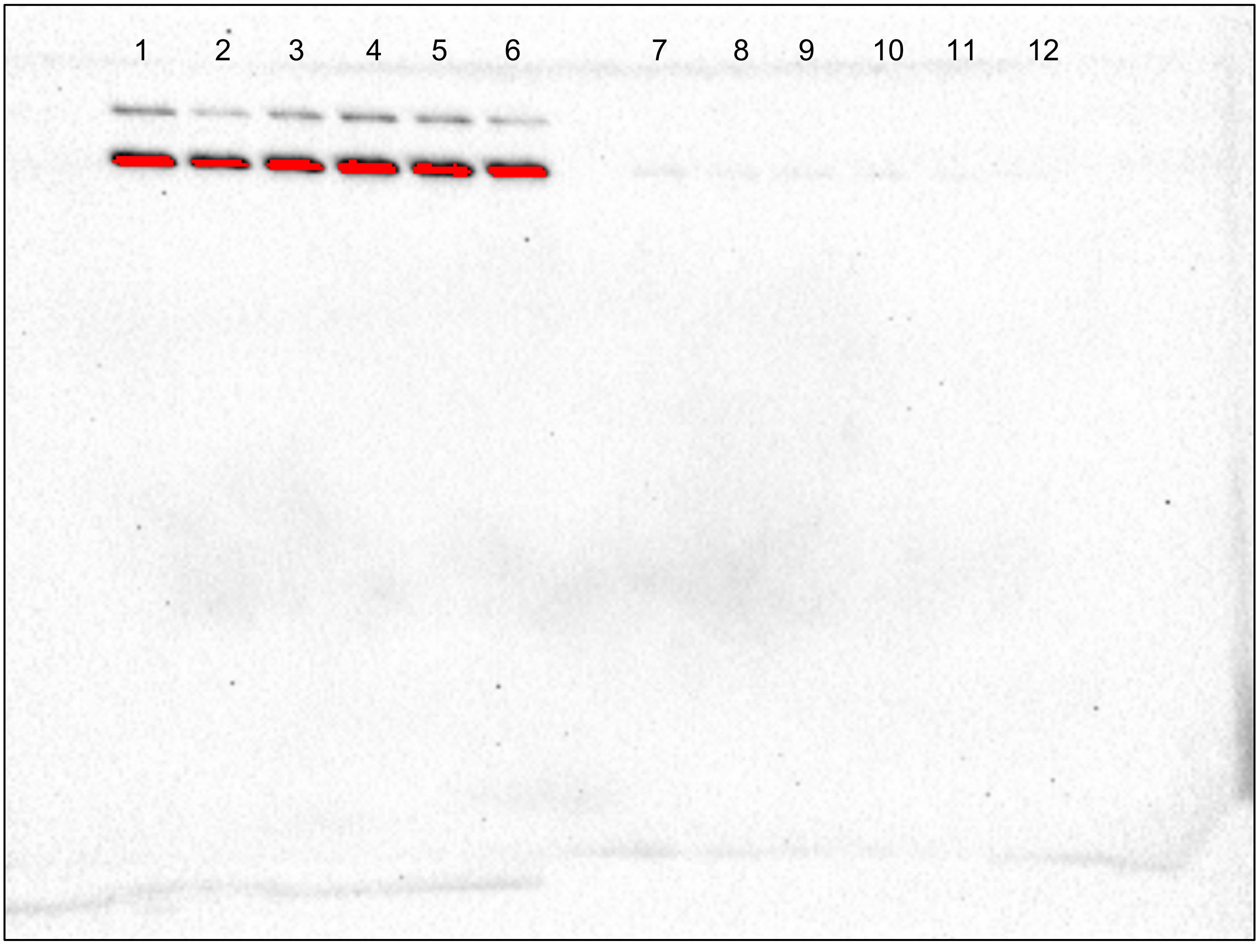

Figure S2C B-actin blot

1. Not included
2. MCF-7 shNT
3. MCF-7 shMETTL3-1
4. MCF-7 shMETTL3-2
5. MCF-7 shMETTL14-1
6. MCF-7 shMETTL14-2
7. MCF-7 shWTAP-1
8. MCF-7 shWTAP-2

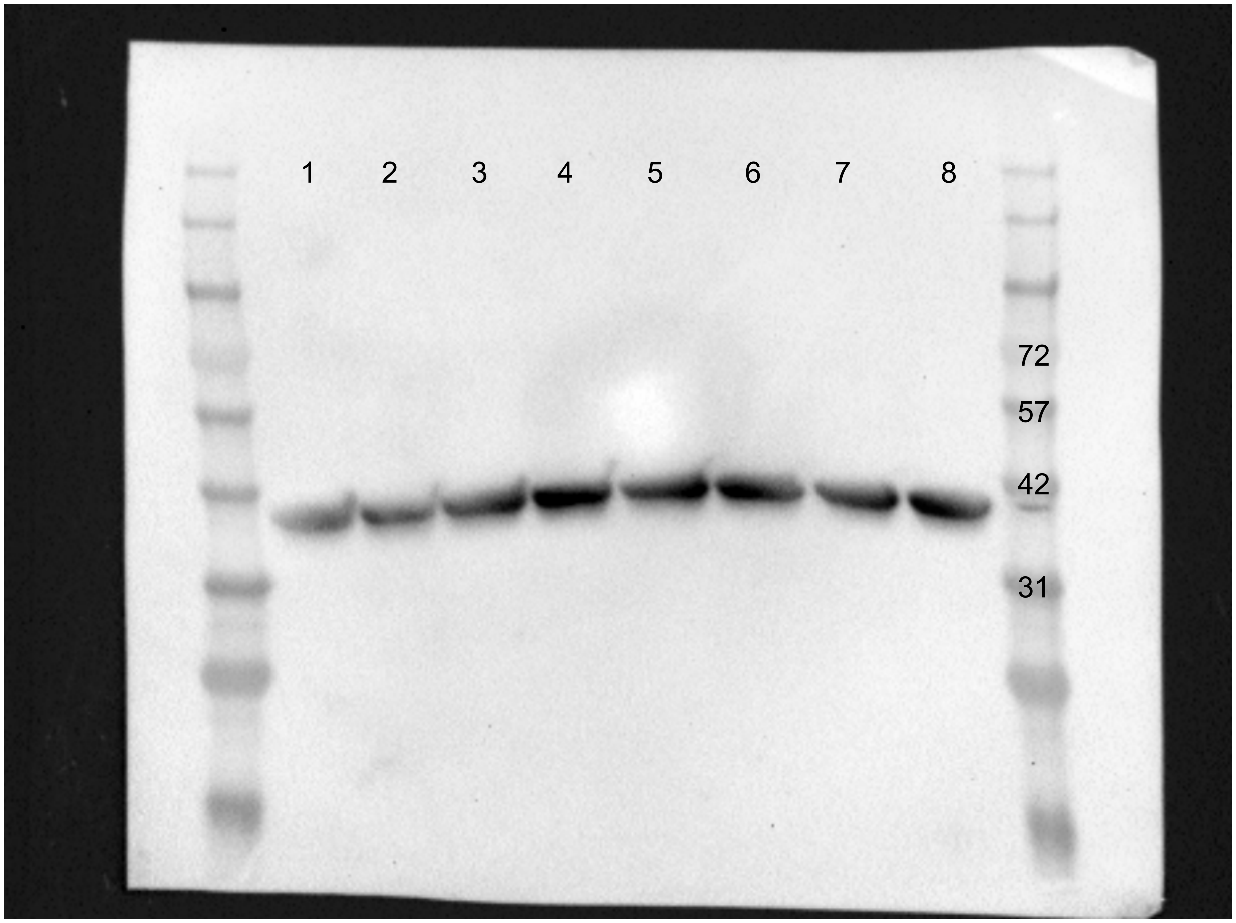

Figure S2C Mettl14 blot

1. Not included
2. MCF-7 shNT
3. MCF-7 shMETTL3-1
4. MCF-7 shMETTL3-2
5. MCF-7 shMETTL14-1
6. MCF-7 shMETTL14-2
7. MCF-7 shWTAP-1
8. MCF-7 shWTAP-2

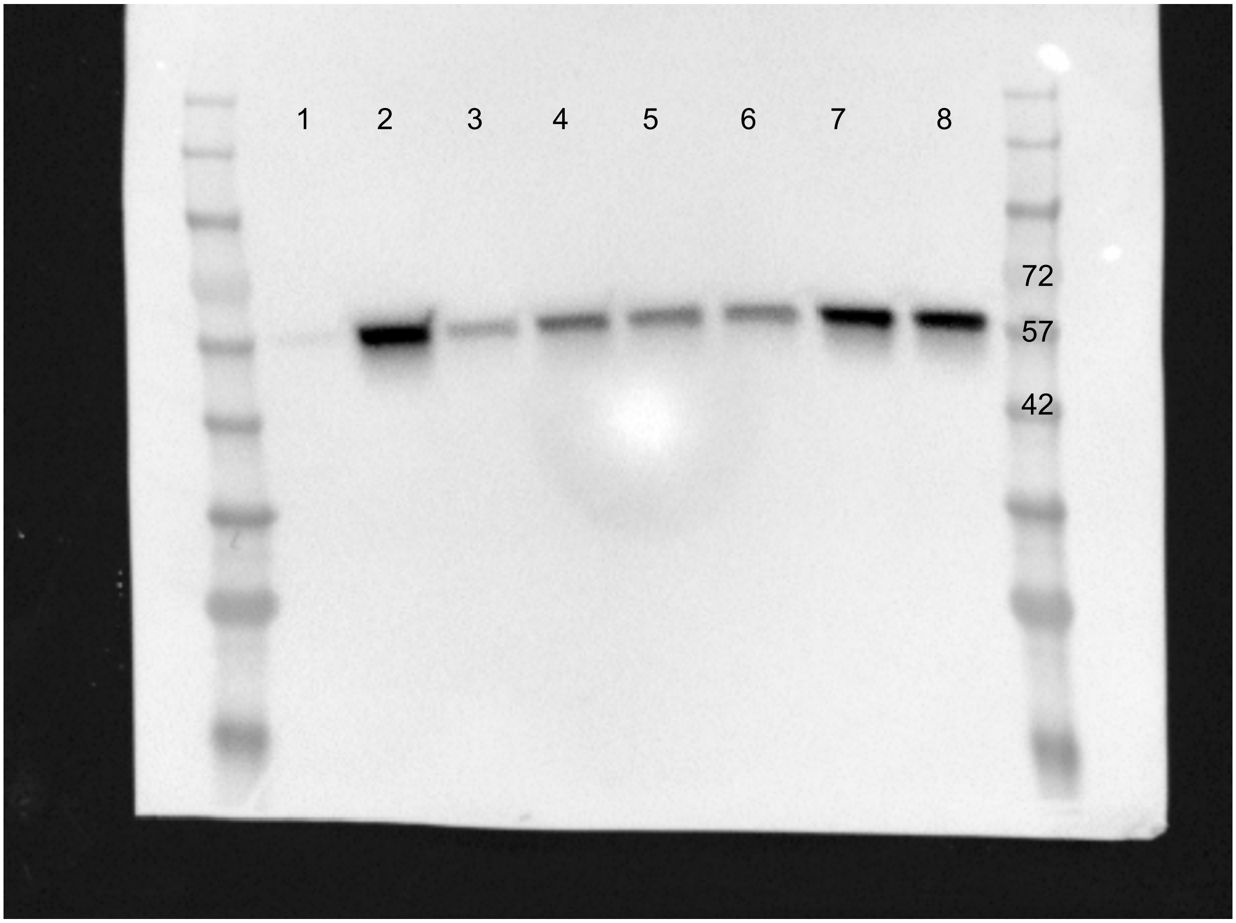

Figure S2C Mettl3 and WTAP blot

1. Not included
2. MCF-7 shNT
3. MCF-7 shMETTL3-1
4. MCF-7 shMETTL3-2
5. MCF-7 shMETTL14-1
6. MCF-7 shMETTL14-2
7. MCF-7 shWTAP-1
8. MCF-7 shWTAP-2

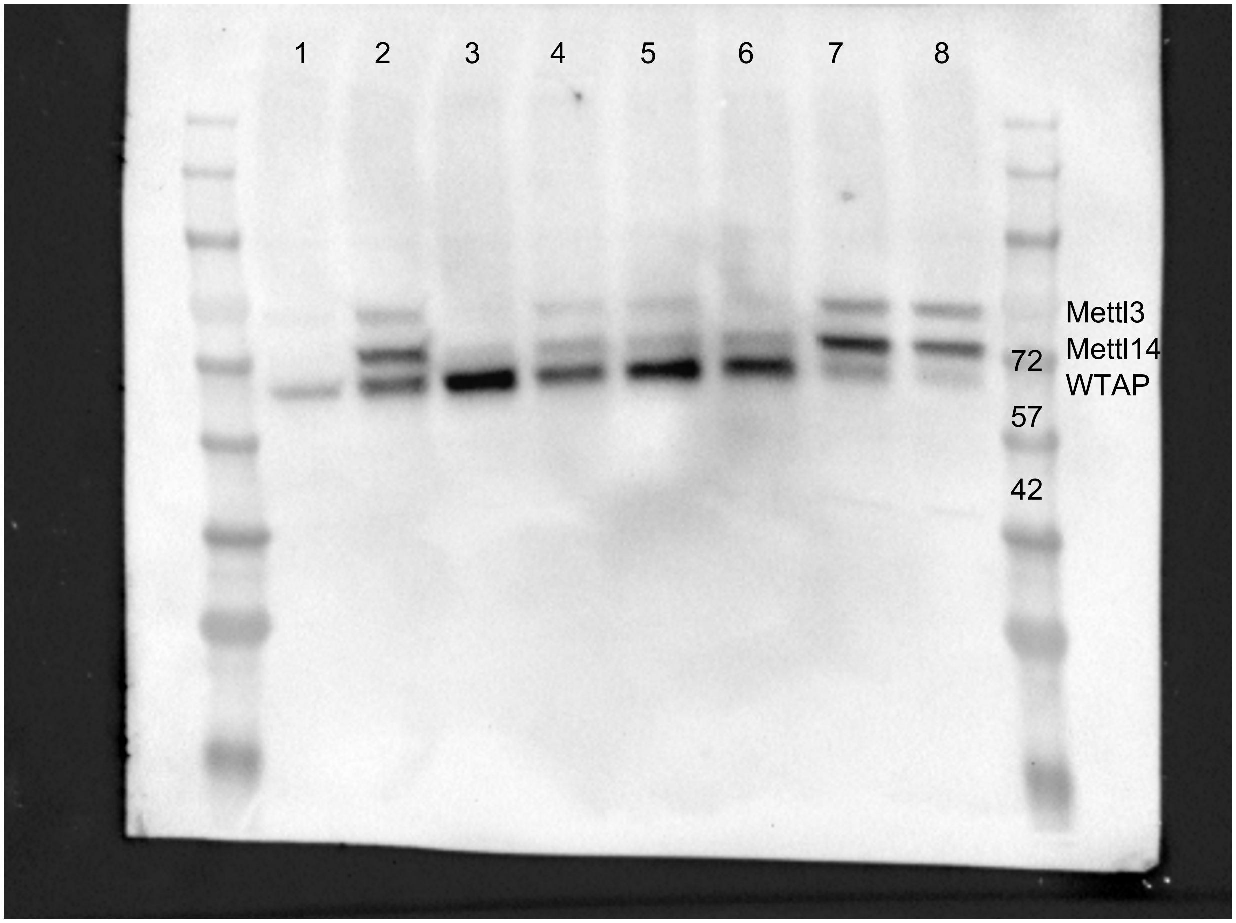

Figure S6A hnRNP A2/B1 blot

Samples:

1. MDA-MB-231 pB-HOTAIR 0.5% Input
2. MDA-MB-231 pB-A783U 0.5% Input
3. MDA-MB-231 pB-AntiLuc 0.5% Input
4. MDA-MB-231 pB-HOTAIR 0.5% Cytoplasm
5. MDA-MB-231 pB-A783U 0.5% Cytoplasm
6. MDA-MB-231 pB-AntiLuc 0.5% Cytoplasm
7. MDA-MB-231 pB-HOTAIR 1% Nucleoplasm
8. MDA-MB-231 pB-A783U 1% Nucleoplasm
9. MDA-MB-231 pB-AntiLuc 1% Nucleoplasm
10. MDA-MB-231 pB-HOTAIR 1% Chromatin
11. MDA-MB-231 pB-A783U 1% Chromatin
12. MDA-MB-231 pB-AntiLuc 1% Chromatin

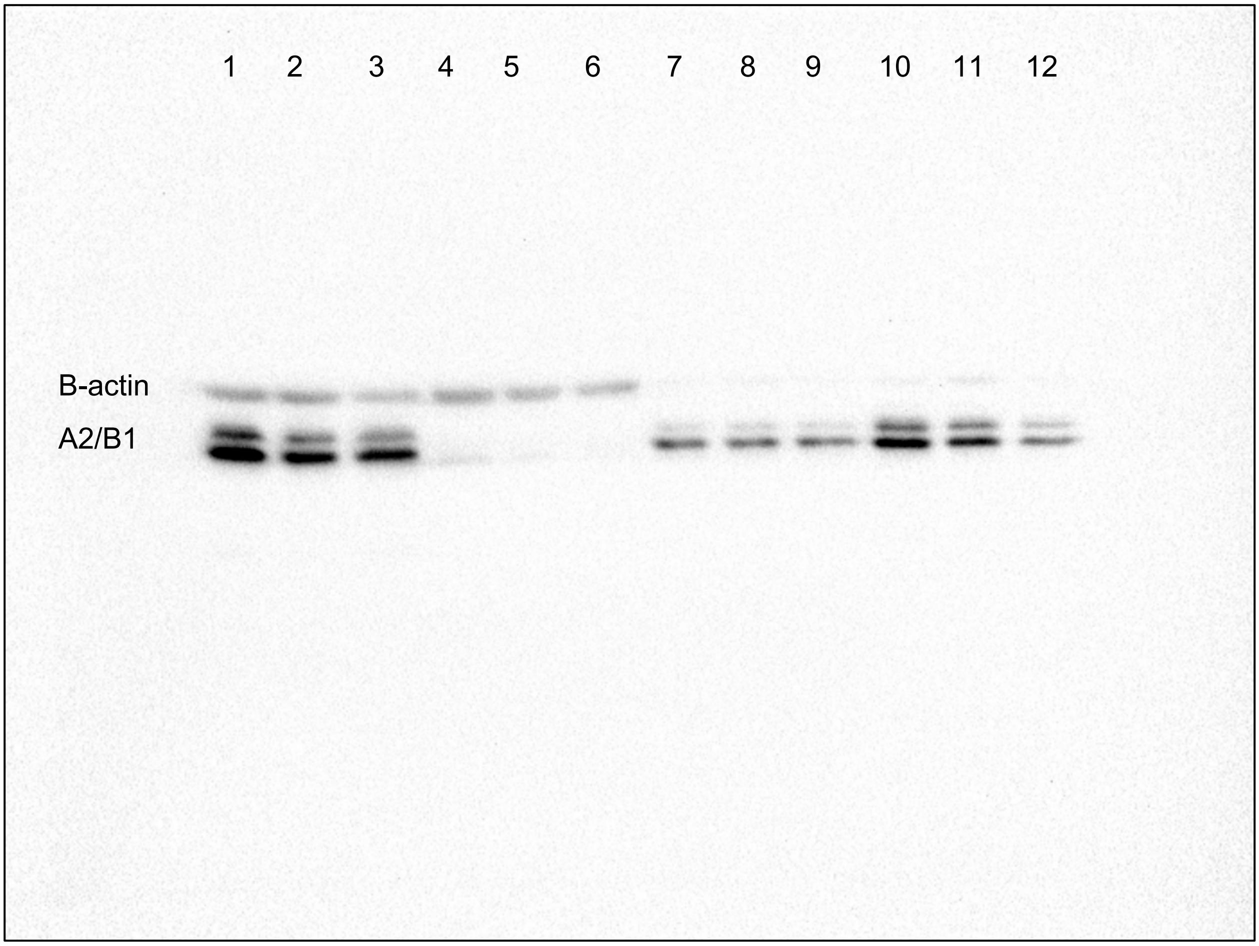

Figure S6A B-actin blot

Samples:

1. MDA-MB-231 pB-HOTAIR 0.5% Input
2. MDA-MB-231 pB-A783U 0.5% Input
3. MDA-MB-231 pB-AntiLuc 0.5% Input
4. MDA-MB-231 pB-HOTAIR 0.5% Cytoplasm
5. MDA-MB-231 pB-A783U 0.5% Cytoplasm
6. MDA-MB-231 pB-AntiLuc 0.5% Cytoplasm
7. MDA-MB-231 pB-HOTAIR 1% Nucleoplasm
8. MDA-MB-231 pB-A783U 1% Nucleoplasm
9. MDA-MB-231 pB-AntiLuc 1% Nucleoplasm
10. MDA-MB-231 pB-HOTAIR 1% Chromatin
11. MDA-MB-231 pB-A783U 1% Chromatin
12. MDA-MB-231 pB-AntiLuc 1% Chromatin

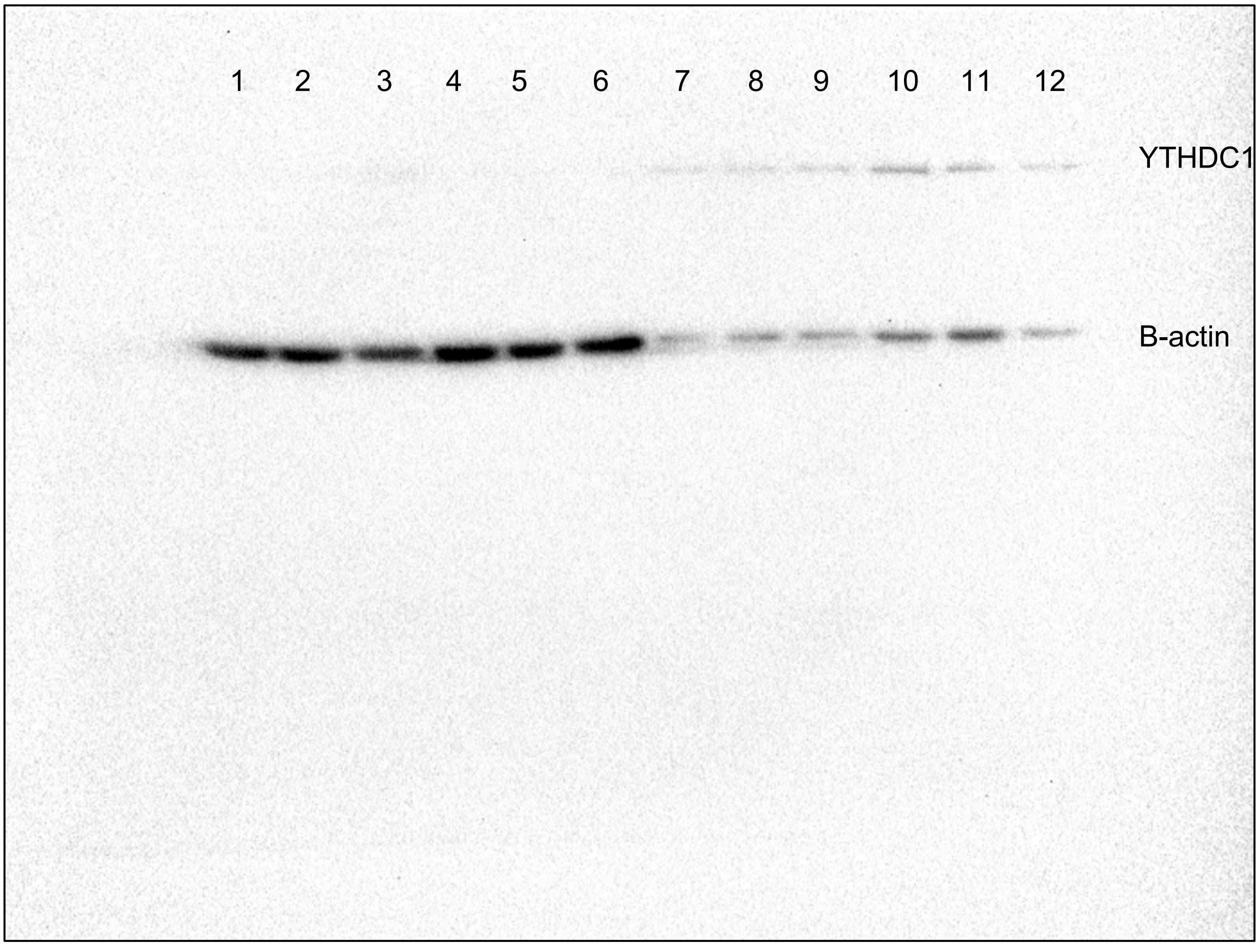

Figure S6A H3 blot

Samples:

1. MDA-MB-231 pB-HOTAIR 0.5% Input
2. MDA-MB-231 pB-A783U 0.5% Input
3. MDA-MB-231 pB-AntiLuc 0.5% Input
4. MDA-MB-231 pB-HOTAIR 0.5% Cytoplasm
5. MDA-MB-231 pB-A783U 0.5% Cytoplasm
6. MDA-MB-231 pB-AntiLuc 0.5% Cytoplasm
7. MDA-MB-231 pB-HOTAIR 1% Nucleoplasm
8. MDA-MB-231 pB-A783U 1% Nucleoplasm
9. MDA-MB-231 pB-AntiLuc 1% Nucleoplasm
10. MDA-MB-231 pB-HOTAIR 1% Chromatin
11. MDA-MB-231 pB-A783U 1% Chromatin
12. MDA-MB-231 pB-AntiLuc 1% Chromatin

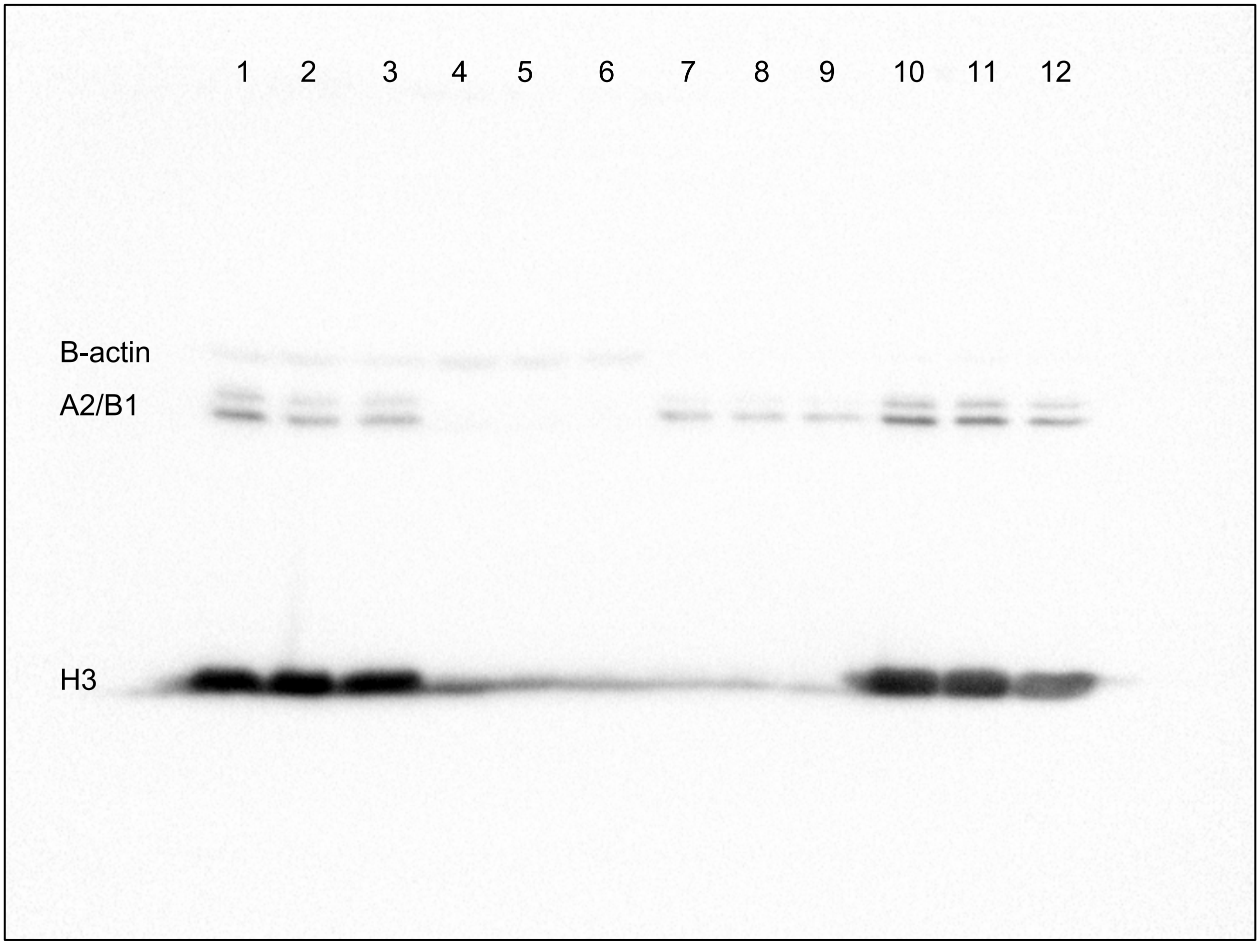

Figure S6A YTHDC1 Blot

Samples:

1. MDA-MB-231 pB-HOTAIR 0.5% Input
2. MDA-MB-231 pB-A783U 0.5% Input
3. MDA-MB-231 pB-AntiLuc 0.5% Input
4. MDA-MB-231 pB-HOTAIR 0.5% Cytoplasm
5. MDA-MB-231 pB-A783U 0.5% Cytoplasm
6. MDA-MB-231 pB-AntiLuc 0.5% Cytoplasm
7. MDA-MB-231 pB-HOTAIR 1% Nucleoplasm
8. MDA-MB-231 pB-A783U 1% Nucleoplasm
9. MDA-MB-231 pB-AntiLuc 1% Nucleoplasm
10. MDA-MB-231 pB-HOTAIR 1% Chromatin
11. MDA-MB-231 pB-A783U 1% Chromatin
12. MDA-MB-231 pB-AntiLuc 1% Chromatin

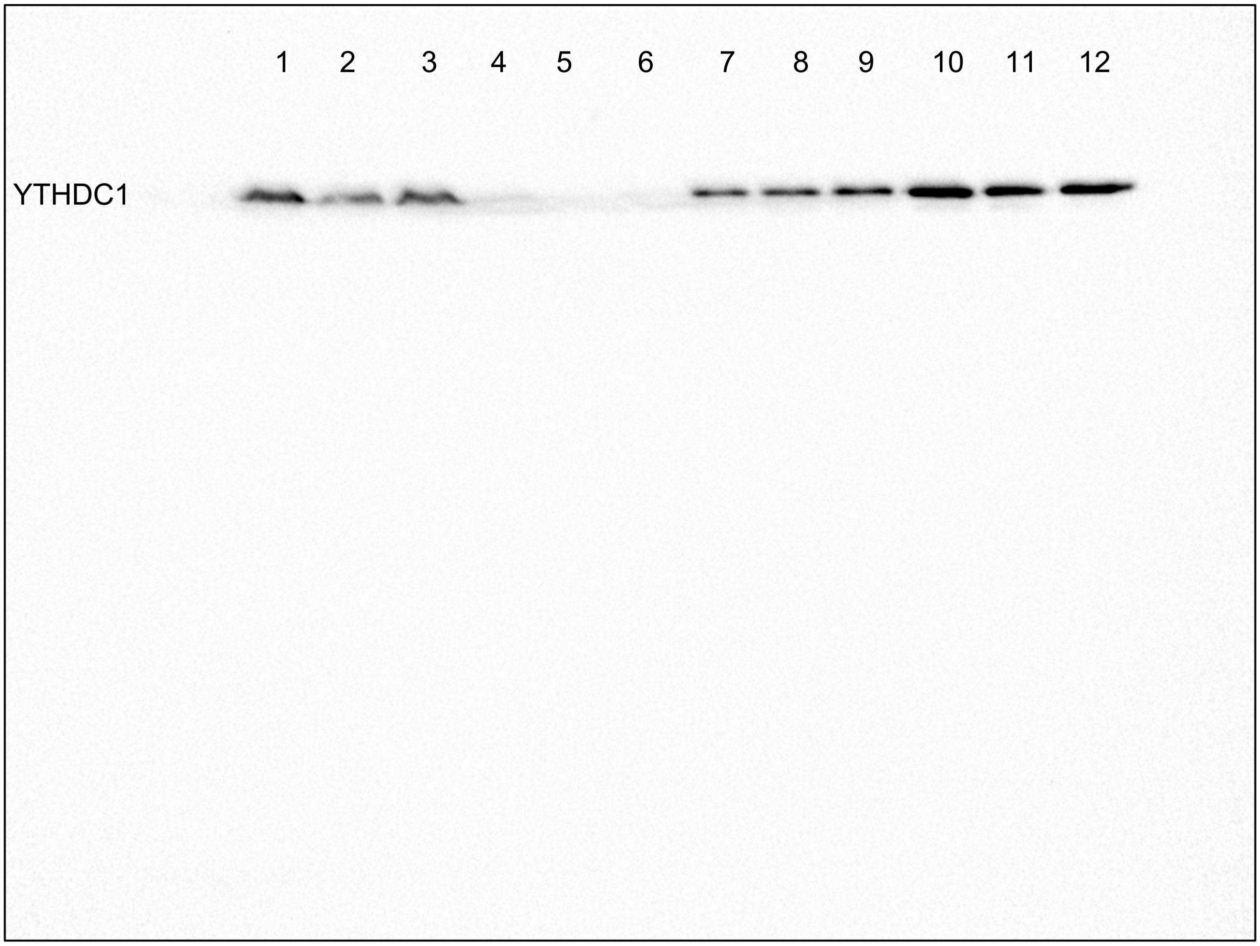

Figure S6B B-actin and hnRNP A2B1 blot (Input + Cytoplasmic)

Samples:
1. MDA-MB-231 pB-HOTAIR pLX-DC1 0.5% Input
2. MDA-MB-231 pB-HOTAIR shNT 0.5% Input
3. MDA-MB-231 pB-HOTAIR shDC1 0.5% Input
4. MDA-MB-231 pB-A783U pLX-DC1 0.5% Input
5. MDA-MB-231 pB-A783U shNT 0.5% Input
6. MDA-MB-231 pB-A783U shDC1 0.5% Input
7. MDA-MB-231 pB-HOTAIR pLX-DC1 0.5% Cytoplasmic
8. MDA-MB-231 pB-HOTAIR shNT 0.5% Cytoplasmic
9. MDA-MB-231 pB-HOTAIR shDC1 0.5% Cytoplasmic
10. MDA-MB-231 pB-A783U pLX-DC1 0.5% Cytoplasmic
11. MDA-MB-231 pB-A783U shNT 0.5% Cytoplasmic
12. MDA-MB-231 pB-A783U shDC1 0.5% Cytoplasmic
13. Not included

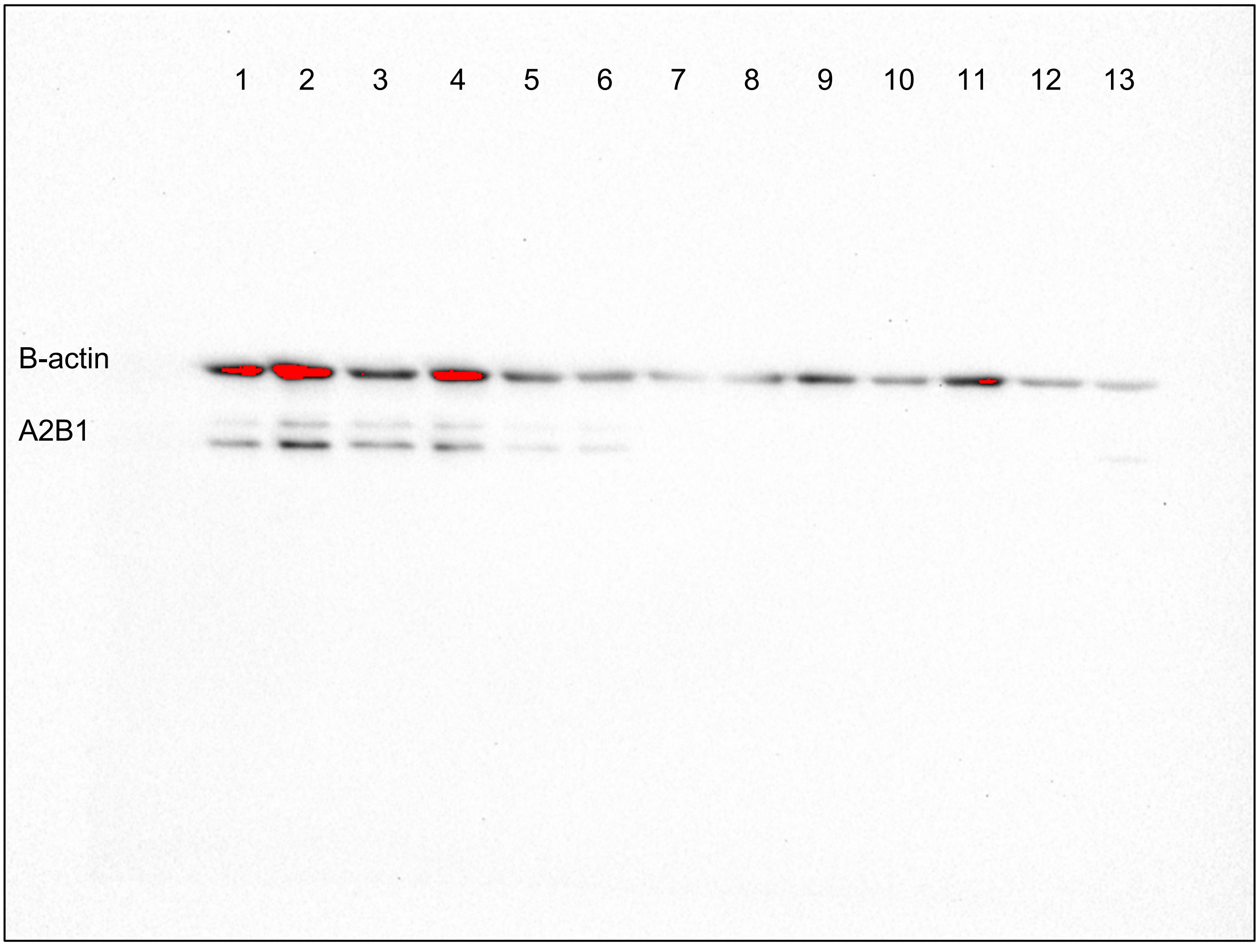

Figure S6B B-actin and hnRNP A2B1 blot (nucleoplasmic + chromatin)

Samples:

1. MDA-MB-231 pB-HOTAIR pLX-DC1 1% nucleoplasmic
2. MDA-MB-231 pB-HOTAIR shNT 1% nucleoplasmic
3. MDA-MB-231 pB-HOTAIR shDC1 1% nucleoplasmic
4. MDA-MB-231 pB-A783U pLX-DC1 1% nucleoplasmic
5. MDA-MB-231 pB-A783U shNT 1% nucleoplasmic
6. MDA-MB-231 pB-A783U shDC1 1% nucleoplasmic
7. MDA-MB-231 pB-HOTAIR pLX-DC1 1% chromatin
8. MDA-MB-231 pB-HOTAIR shNT 1% chromatin
9. MDA-MB-231 pB-HOTAIR shDC1 1% chromatin
10. MDA-MB-231 pB-A783U pLX-DC1 1% chromatin
11. MDA-MB-231 pB-A783U shNT 1% chromatin
12. MDA-MB-231 pB-A783U shDC1 1% chromatin

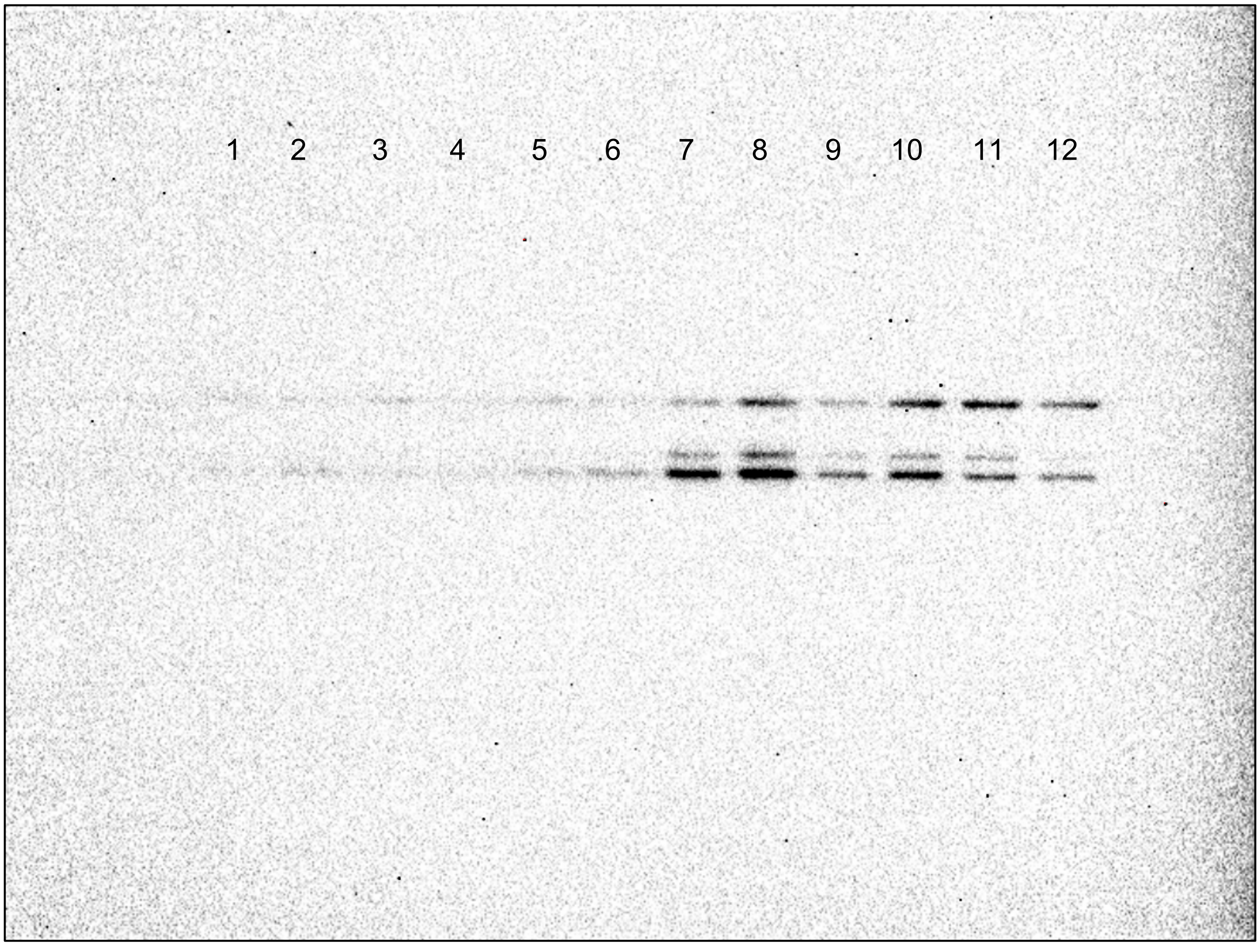

Figure S6B H3 blot (Input + Cytoplasmic)

Samples:

1. MDA-MB-231 pB-HOTAIR pLX-DC1 0.5% Input
2. MDA-MB-231 pB-HOTAIR shNT 0.5% Input
3. MDA-MB-231 pB-HOTAIR shDC1 0.5% Input
4. MDA-MB-231 pB-A783U pLX-DC1 0.5% Input
5. MDA-MB-231 pB-A783U shNT 0.5% Input
6. MDA-MB-231 pB-A783U shDC1 0.5% Input
7. MDA-MB-231 pB-HOTAIR pLX-DC1 0.5% Cytoplasmic
8. MDA-MB-231 pB-HOTAIR shNT 0.5% Cytoplasmic
9. MDA-MB-231 pB-HOTAIR shDC1 0.5% Cytoplasmic
10. MDA-MB-231 pB-A783U pLX-DC1 0.5% Cytoplasmic
11. MDA-MB-231 pB-A783U shNT 0.5% Cytoplasmic
12. MDA-MB-231 pB-A783U shDC1 0.5% Cytoplasmic
13. Not included

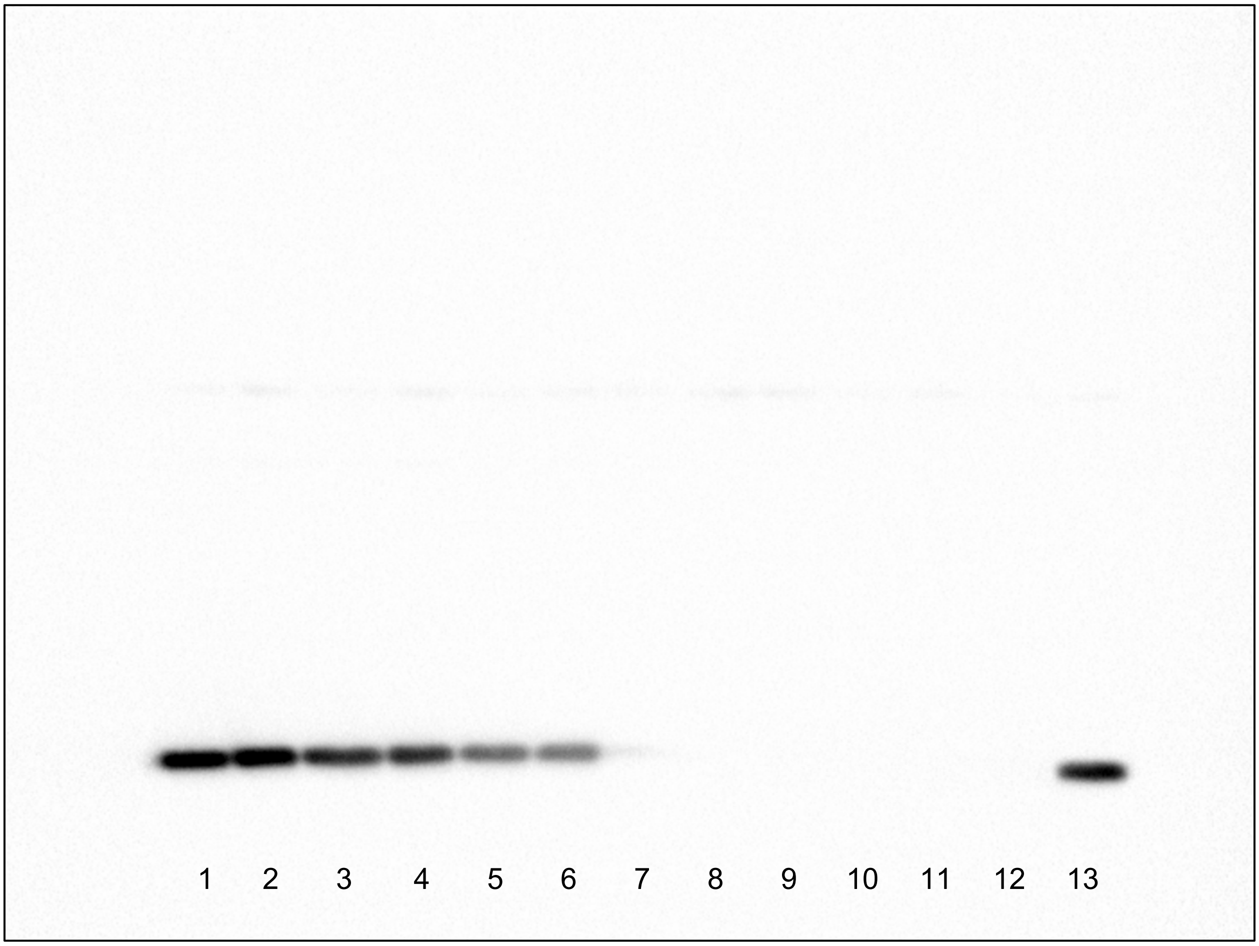

Figure S6B H3 blot (nucleoplasmic + chromatin)

Samples:

1. MDA-MB-231 pB-HOTAIR pLX-DC1 1% nucleoplasmic
2. MDA-MB-231 pB-HOTAIR shNT 1% nucleoplasmic
3. MDA-MB-231 pB-HOTAIR shDC1 1% nucleoplasmic
4. MDA-MB-231 pB-A783U pLX-DC1 1% nucleoplasmic
5. MDA-MB-231 pB-A783U shNT 1% nucleoplasmic
6. MDA-MB-231 pB-A783U shDC1 1% nucleoplasmic
7. MDA-MB-231 pB-HOTAIR pLX-DC1 1% chromatin
8. MDA-MB-231 pB-HOTAIR shNT 1% chromatin
9. MDA-MB-231 pB-HOTAIR shDC1 1% chromatin
10. MDA-MB-231 pB-A783U pLX-DC1 1% chromatin
11. MDA-MB-231 pB-A783U shNT 1% chromatin
12. MDA-MB-231 pB-A783U shDC1 1% chromatin

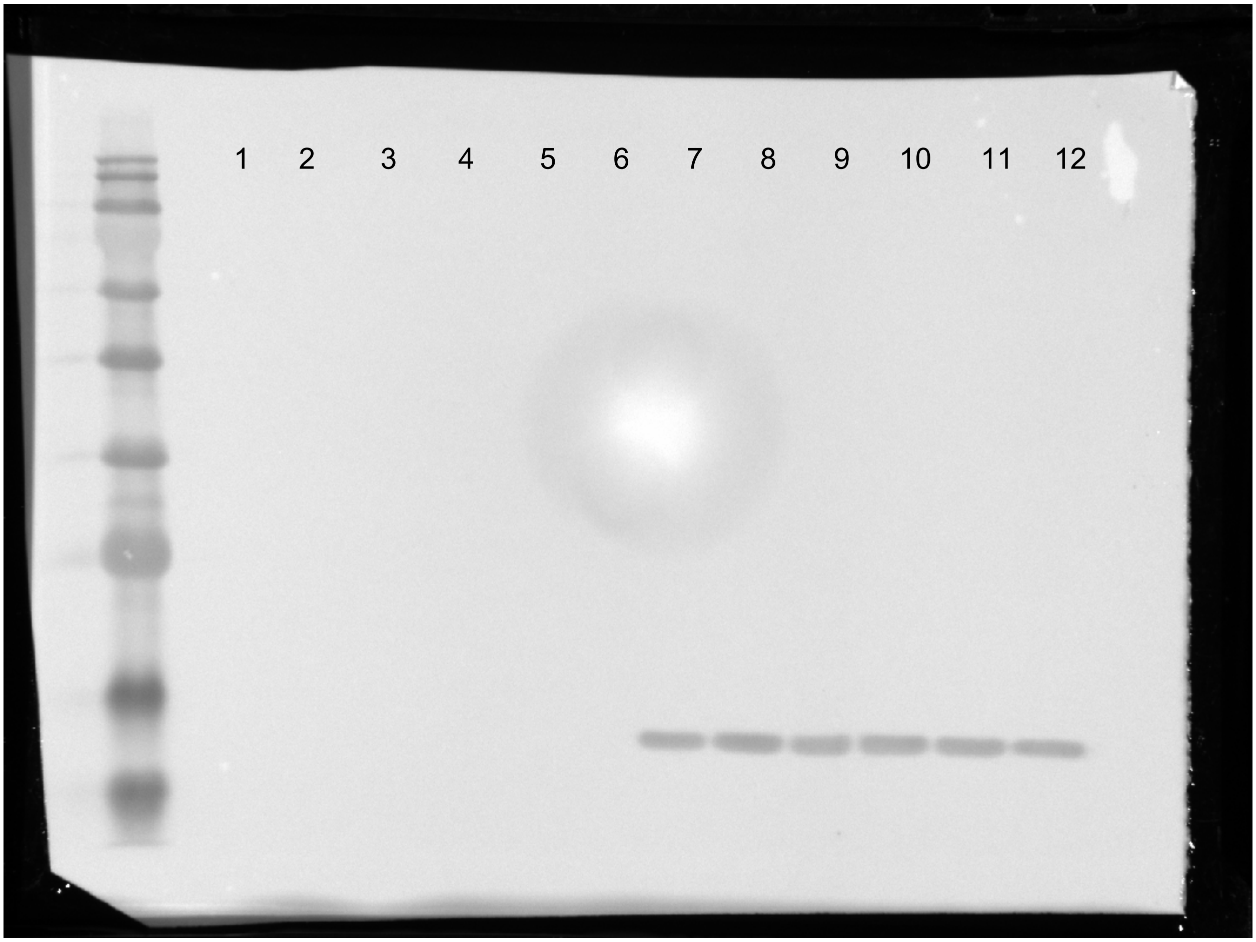

Figure S6B YTHDC1 blot (Input + Cytoplasmic)

Samples:

1. MDA-MB-231 pB-HOTAIR pLX-DC1 0.5% Input
2. MDA-MB-231 pB-HOTAIR shNT 0.5% Input
3. MDA-MB-231 pB-HOTAIR shDC1 0.5% Input
4. MDA-MB-231 pB-A783U pLX-DC1 0.5% Input
5. MDA-MB-231 pB-A783U shNT 0.5% Input
6. MDA-MB-231 pB-A783U shDC1 0.5% Input
7. MDA-MB-231 pB-HOTAIR pLX-DC1 0.5% Cytoplasmic
8. MDA-MB-231 pB-HOTAIR shNT 0.5% Cytoplasmic
9. MDA-MB-231 pB-HOTAIR shDC1 0.5% Cytoplasmic
10. MDA-MB-231 pB-A783U pLX-DC1 0.5% Cytoplasmic
11. MDA-MB-231 pB-A783U shNT 0.5% Cytoplasmic
12. MDA-MB-231 pB-A783U shDC1 0.5% Cytoplasmic
13. Not included

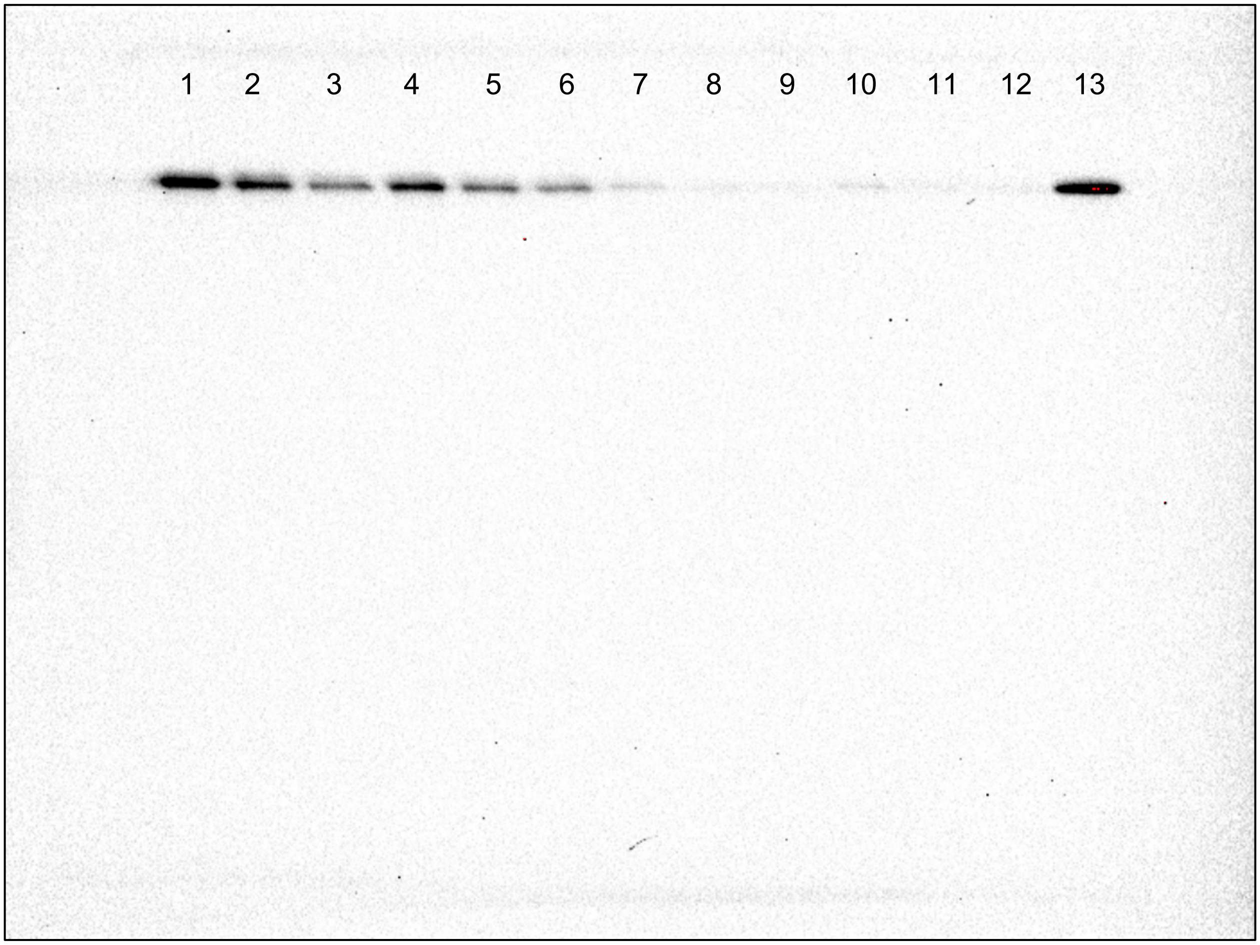

Figure S6B B-actin and hnRNP A2B1 blot (nucleoplasmic + chromatin)

Samples:

1. MDA-MB-231 pB-HOTAIR pLX-DC1 1% nucleoplasmic
2. MDA-MB-231 pB-HOTAIR shNT 1% nucleoplasmic
3. MDA-MB-231 pB-HOTAIR shDC1 1% nucleoplasmic
4. MDA-MB-231 pB-A783U pLX-DC1 1% nucleoplasmic
5. MDA-MB-231 pB-A783U shNT 1% nucleoplasmic
6. MDA-MB-231 pB-A783U shDC1 1% nucleoplasmic
7. MDA-MB-231 pB-HOTAIR pLX-DC1 1% chromatin
8. MDA-MB-231 pB-HOTAIR shNT 1% chromatin
9. MDA-MB-231 pB-HOTAIR shDC1 1% chromatin
10. MDA-MB-231 pB-A783U pLX-DC1 1% chromatin
11. MDA-MB-231 pB-A783U shNT 1% chromatin
12. MDA-MB-231 pB-A783U shDC1 1% chromatin

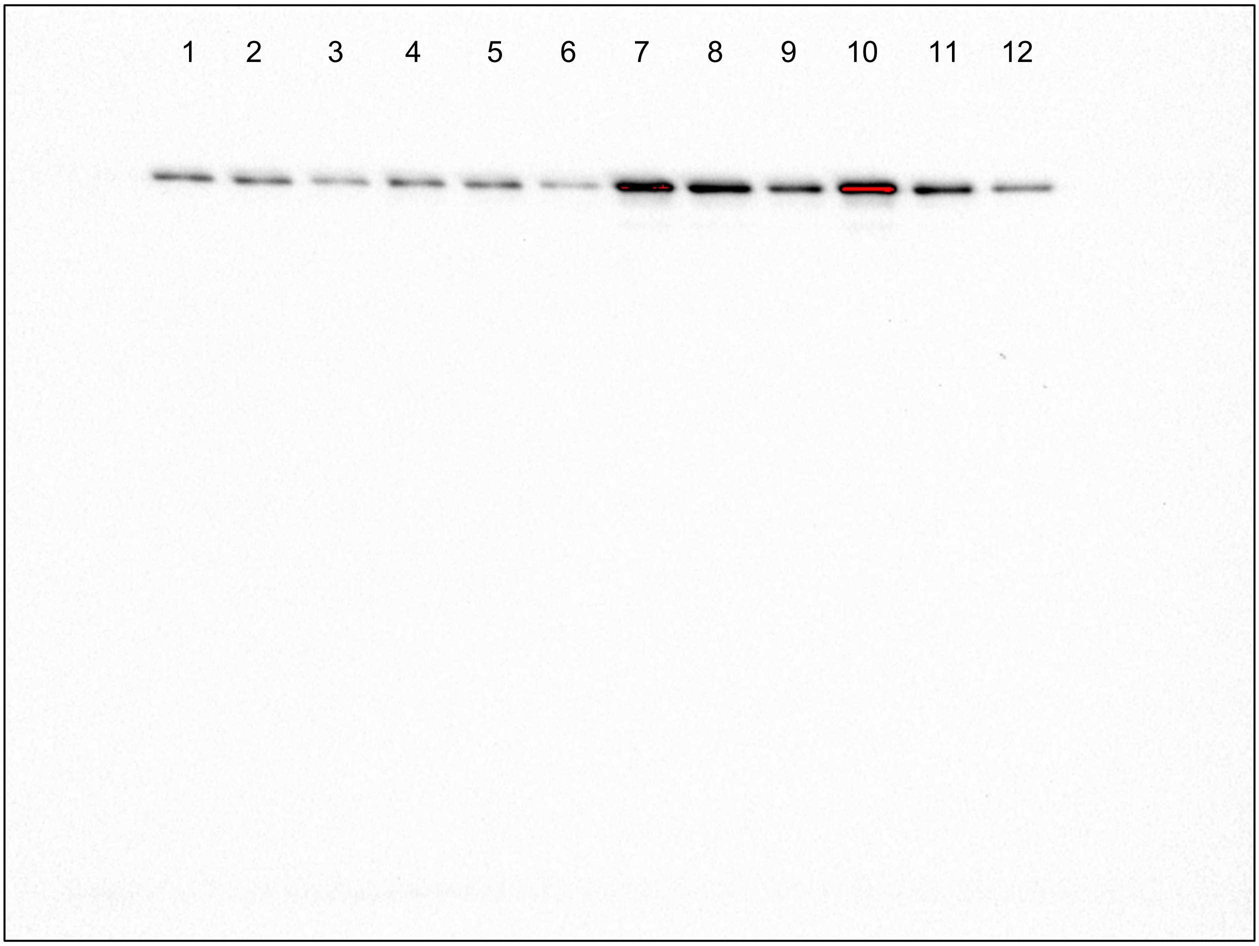

Supplement: S1 Raw Images — (PDF) [file pbio.3001885.s020.pdf]
