# Supplementary material for: A single N6-methyladenosine site regulates lncRNA HOTAIR function in breast cancer cells
Source: PLoS Biol. 2022 Nov 28;20(11):e3001885. doi: 10.1371/journal.pbio.3001885 (PMC9731500; doi:10.1371/journal.pbio.3001885)
Supplement: Attachment — Submitted filename: PormanEtAl_ResponsetoReviewers_V2.pdf [file pbio.3001885.s021.pdf]

We thank the reviewers for their thorough reading of our manuscript, for the enthusiasm regarding its impact, and the helpful suggestions. We have revised the manuscript significantly, including new data and text where appropriate to address the reviewers' points. Our manuscript with highlighted changes in green text is uploaded, which is the best file for checking the specific passages referenced by line in our responses below (this version also has higher-quality figures in the PDF).

The manuscript is much improved and provides for an even clearer conclusion of this important methylation event in HOTAIR, how it dictates the molecular and cellular functions of the lncRNA, and what happens when this is prevented. We hope the reviewers agree that the manuscript is sufficiently improved to warrant publication in its current form.

Below are point-by-point responses to the reviews, with our response in blue text.

REVIEWS:

Reviewer #1: In this manuscript, Porman et al investigate the role of m6A in HOTAIR-mediated transcriptional repression. The authors report that methylation, primarily of a single residue, results in chromatin association in a manner mediated by YTHDC1, which culminates in HOTAIR stabilization, transcriptional repression, increased proliferation, and potentially reduced survival in cancer patients. There are multiple dimensions of this manuscript that I enjoyed. This work is among the few in literature where a rigorous attempt is made to connect m6A at a single position to both a molecular and a physiological outcome. Also, the depth of mechanistic examination performed by the authors is impressive. The manuscript is also well and concisely written, and I enjoyed the fact that at various instances where observations were inconsistent with their model, the authors explicitly pointed this out and discussed it.
Nonetheless, there remain points of lingering concern, where both clarifications and additional experimentation could help substantiate and consolidate the results.

Major points:

1. Our first concern pertains to the presence of m6A at the position reported by the authors. As also noted by the authors, the position that they detect is an atypical one - it lacks a classical 'DRACH' consensus motif, and as such would be predicted to not undergo modifications at all, or at low stoichiometries. This position was exclusively (yet consistently) identified on the basis of eCLIP experiments on WT samples. Such eCLIP experiments were not performed following knockdown of methyltransferase machinery components. Moreover, the authors report no change in HOTAIR immunoprecipitation efficiency using an anti-m6A antibody in A783U mutants lacking m6A at this position (we note that the eCLIP signature is lost in this mutant). Given the centrality of the claims concerning m6A at this position, in our view it is critical that this point be confirmed both on the basis of additional controls (e.g. eliminating methyltransferase components) and ideally also approaches.

We appreciate the reviewer's suggestion to better highlight the evidence that site A783 is a genuine METTL3/14 target. While our original manuscript did describe the METTL3, METTL14, and WTAP knockdown experiments in MCF7 cells, we neglected to highlight in the text that this is a condition where A783 is the only modified site detected in HOTAIR. We observe a 3-5-fold loss of meRIP efficiency of HOTAIR in these knockdowns, which is consistent with the canonical mRNA methylation machinery targeting HOTAIR A783. As for no decrease in meRIP in A783U mutants in MDA-MB-231 cells where it is

overexpressed, this is in a context where we know HOTAIR bears other m6A sites that are unchanged by A783U mutation, including one ~10 nt away, A772, making it unlikely that the IP would discriminate a loss of methylation at A783. The text is revised to highlight the importance of the MCF7 knockdown findings (Lines 126-131) and to explain the A783U results (Lines 150-153).

While we recognize that the 'DRACH' consensus motif is the most commonly m6A-modified motif, we note that previous studies have found that the core 'RAC' motif is the minimal required sequence for m6A methylation in human cells (Wei & Moss, *Biochemistry* 1977; Meyer et al. *Cell* 2012; Linder et al. *Nat. Prot.* 2015; Ke et al. *G&D*, 2016).

2. An additional major concern is that a large body of the conclusion rests on a single mutant (A783U). While the tacit assumption is that this particular mutant differs only in methylation status, it cannot be ruled out - in particular for a structured molecule such as HOTAIR - that this particular mutation impacts the structure or folding of HOTAIR, which underlies some (or all) observations. The conclusions would be hugely boosted by introducing two additional mutants, perturbing the 'C' immediately downstream of the methylated site and the 'A' upstream, and demonstrating that these mutants phenocopy the A783U mutants in growth and chromatin localization.

We appreciate the reviewer's concerns about the potential for off-target structural effects of this single nucleotide mutation. However, the location of this nucleotide is in a single-stranded region of HOTAIR according to previous structural studies (Somarowthu *et al.* Mol Cell 2015). Additionally, the methyltransferase does not readily methylate adenosine residues in highly structured RNA regions (Meiser, Mench, & Hengesbach. (2020) *Biological chemistry*, 402(1), 89–98.) Therefore, the mutation of A783 is unlikely to cause loss of secondary structure important for HOTAIR function. Considering this, we decided that making additional mutations of the same position and performing the many experiments we have done for A783U would not provide additional conclusiveness. We do feel that the tethering experiments of Figure 6 squarely point to methylation and recognition by YTHDC1 being the important mechanism that is disrupted by the A783U mutant. However, since we cannot entirely rule out a potential, albeit unlikely, unintended structural change, we have noted this in the discussion (Lines 442-446).

More minor points:

3. An aspect I found somewhat confusing in this manuscript, and which also renders the interpretation of many of the experiments confusing, is the causal relationship between YTHDC1 and HOTAIR expression levels. In Figure 1D the authors demonstrate that mutant HOTAIR gives rise to reduced levels of YTHDC1 than WT HOTAIR, suggesting that YTHDC1 is stabilized by HOTAIR. Yet, the model argued by the authors - and for which they also display evidence - is that YTHDC1 is stabilized by HOTAIR. So is there a circular relationship here? This should be clarified. Related to this aspect, we found it confusing that if these genes are positively correlated with another, and increased levels of both genes are required for increased proliferation (Figure 2E), then how does this connect to the Kaplan Meier curves, according to which the genes appear to display an opposite effect on survival (for YTHDC1, reduced levels are associated with poorer survival, for HOTAIR with improved survival)? More generally, we did not feel that this section (Figure 3) was very compelling. In general, the associations with gene expression levels were weak, and the differences in association with HOTAIR between high YTHDC1 and low YTHDC1

were, again, weak. We do not think these are of critical importance for the manuscript and leave this to the author's discretion.

We thank the reviewers for highlighting the places where we can more clearly describe the relationship between HOTAIR and YTHDC1. We believe the reviewer is describing Figure 2C and 2D of the original manuscript, where a ~50% reduction in YTHDC1 is observed with A783U mutant. This provides some evidence that the thousands of copies of HOTAIR with methylated A783 might provide some stability to YTHDC1 when it binds there. We have edited the sections describing this to make it more clear of this potential relationship of mutual stabilization, with our model being that the protein-RNA interaction itself dictates this, since we have no evidence that HOTAIR regulates YTHDC1 gene expression.

We agree with the reviewer that the clinical outcome of YTHDC1 mRNA expression levels is somewhat confusing, so have removed this more extensive analysis from the text. We also note the clinical differences observed for YTHDC1 mRNA and protein expression (Figure S4C-D), which we discuss in the results section (Lines 250-257). Due to these confounding factors, and the fact that it is difficult to examine the relationship between YTHDC1 protein levels and HOTAIR RNA levels (to our knowledge, no available breast cancer studies report on both protein and RNA abundance while following outcomes), we have decided to remove Figure 3 and condense the clinical data into Figure S4.

4. I loved the experiments in Figure 4E-F dissecting chromatin localization of WT and mutant HOTAIR following perturbation of YTHDC1 levels, and the results for the mutant seem very compelling. However, I find it difficult to accept the explanation by the authors for the complete lack of effect of YTH depletion on WT HOTAIR. A key component of their model is that YTHDC1 binding to A783 of HOTAIR gives rise to chromatin localization. Yet, in this key experiment, depletion of YTHDC1 gives rise to even slightly increased chromatin localization. The authors suggest this could have to do with insufficient knockdown, yet clearly the levels of knockdown are sufficient to destabilize HOTAIR and to give rise to phenotypes. Could this possibly be a normalization issue, potentially caused by the reduced levels of HOTAIR following the knockdown? Assuming it is the knockdown efficiencies, can the authors perturb YTHDC1 using other means, and confirm that under this scenario chromatin localization is reduced?

We thank the reviewer for the appreciation of our work in Figure 4E-F (now 3C-E), this was a critical first step in understanding how interaction of YTHDC1 with methylated A783 contributes to gene regulation. As the reviewer suggested, the lack of effect of YTHDC1 depletion on WT HOTAIR chromatin localization can be explained by the normalization. Upon YTHDC1 knockdown, HOTAIR levels decrease due to the decrease in YTHDC1 binding. Our evidence suggests that sites other than A783 are affected, potentially due to lower occupancy/affinity than A783, because A783U HOTAIR levels are also decreased upon YTHDC1 knockdown. But the remaining WT HOTAIR is still DC1-bound, and we propose this occurs at the high affinity m6A783, and therefore remains localized to chromatin. Thus, the fraction of HOTAIR that is chromatin localized in this context is similar to WT, although the overall levels of HOTAIR are greatly reduced in this context. We have added additional explanation to the results section to clarify this (Lines 275-291).

5. In Figure 5 the authors seek to draw a direct connection between HOTAIR and YTHDC1-mediated transcriptional repression using a reporter construct. Yet, in these experiments the authors omitted the control of the A783U mutant. While this result therefore establishes some requirement of YTHDC1 in mediating repression, it fails to associate this with HOTAIR methylation.

This system was built by a different group who kindly provided the reagents. We are limited in our ability to construct a similar cell line expressing the A783U HOTAIR due to lack of appropriate parental cell lines. To further associate this effect with HOTAIR methylation, we have now performed knockdown of METTL3, the enzymatic component of the m6A methyltransferase complex, and confirmed a similar increase in luciferase expression. This work has been added to Figure 4. In general, the support for YTHDC1-HOTAIR interaction and the role of m6A783 is provided extensively in other experiments included in the paper. Our main goal in this set of experiments is to highlight the outcome to transcriptional regulation when HOTAIR and DC1 associate via m6A. Our subsequent gene expression analysis demonstrated that disruption of A783 methylation is sufficient to ablate most gene repression while restoring YTHDC1 at this site is sufficient to restore repression. We hope the further experiments and explanation of our evidence and technical limitations is sufficient response.

6. Finally, the tethering experiments conducted in Figure 7 provide substantial support for the model proposed by the authors, demonstrating that chromatin association of a HOTAIR mutant can be rescued by tethering it to YTHDC1. It is unclear to me, though, why in these experiments no stabilization of HOTAIR is observed. In addition, can the proliferation phenotype - that is lost in the A783U mutant - also be rescued in these experiments, which would elegantly combine the molecular and physiological levels?

We thank the reviewer and agree that the tethering experiments are very powerful. We have performed additional experiments to examine the proliferation and invasion in cells that have YTHDC1 tethered to HOTAIR. These findings have been added to Figure 6 and reveal that tethering DC1 to A783U HOTAIR mediates a shorter doubling time (6E) and an increase in invasion (6F). These assays were more challenging, both due to a higher reliance on transfection efficiency to affect the cell biology in a short period of transient transfection and due to the early points after transfection itself perturbing the cells. Nevertheless, the data support our model where DC1 interaction at A783 mediate chromatin interactions and promotion of proliferation and invasion by HOTAIR.

To address the first part of this point, our model proposes that m6A at A783 is not required for stability of HOTAIR, but that HOTAIR stability is mediated by other m6A sites in the HOTAIR transcript (see Figure 4H). We do not see a measurable loss of steady-state RNA levels with the A783 mutant (Figure 1D). Therefore, in the context of WT and A783U HOTAIR, tethering DC1 near nt783 does not enable further stabilization of HOTAIR, as other m6A sites are still present and can interact with DC1 to mediate its stabilization.

Reviewer #2: The manuscript titled "A single N6-methyladenosine site in lncRNA HOTAIR regulates its function in breast cancer cells" by Porman et al. reports the finding that a particular m6A modification site on HOTAIR is important for proliferation and invasion of triple-negative breast cancer cells. The authors present data to suggest a mechanistic model where YTHDC1 is the reader that is recruited to the particular methylation site (A783) and that it impacts chromatin association and expression of HOTAIR. Overall, the big-picture model that a single m6A site on HOTAIR can impact gene expression is supported well and interesting. Here are some comments and suggestions for revising the manuscript.

1. While the importance of A783 is clear, the evidence for YTHDC1 to be singularly playing a role in HOTAIR-mediated breast cancer progression is not as strong. YTHDC1 binds most m6A-modified RNAs, and RIP procedure might result in associations that do not occur in the cell (eg. due to different localization). Is there evidence that YTHDC1 binds A783 in cells?

We thank the reviewer for highlighting this question. We have included additional language and experiments to address this. Several lines of evidence support our conclusion that YTHDC1 binds m6A-modified A783 in cells:

1) YTHDC1 binds to HOTAIR in MCF-7 cells (Figure 2A), and A783 is the only m6A site detected in this context (Table S1). We note this more explicitly in the text (see Line 201-206).
2) New data: YTHDC1 has increased association with the region of HOTAIR containing m6A783 in MCF-7 cells (Figure 2D).
3) Additional revised data analysis: YTHDC1 interaction with HOTAIR is significantly reduced in cells that express A783U mutant HOTAIR (Figure 2C).

We believe that these data provide strong support for YTHDC1 interaction with m6A modified A783 of HOTAIR and support the results of the *in vitro* RNA pulldown experiment.

2. YTHDC1 seems to have a relationship with HOTAIR, given the expression level changes. Do the authors see no such relationship for other readers in the YTH-family?

While we have not performed extensive analysis on overexpression or knockdown of other YTH family members, we have performed RNA immunoprecipitation experiments with YTHDF1 and YTHDF2 in MCF-7 cells. Here, we observed a general non-specific binding across the HOTAIR transcript (Supplementary Figure 2D-E). This contrasts with YTHDC1 which demonstrates enriched binding at m6A783 of HOTAIR (Figure 2D). This supports the role of methylation of A783 acting as a switch to promote YTHDC1-specific mechanisms.

While we cannot rule out a role for other YTH family members in regulating HOTAIR, this study focuses on the nuclear roles of HOTAIR m6A in regulating gene expression, therefore cytoplasmic readers YTHDF1-3 are not in the scope of this specific mechanism. The fact that rescue of A783U mutation by YTHDC1 tethering (experiments in Figure 6) restores HOTAIR-dependent activities affected by the mutant also supports the focus on YTHDC1.

3. The in vitro experiments to show that YTHDC1 binds A783 of HOTAIR also have some issues. How did the authors confirm that METTL3/14 methylate HOTAIR at the correct position (A783) in vitro?

In vitro transcripts for methylation were prepared identically to structural HOTAIR studies performed by Somarowthu *et al.* (2015) and they have determined that this site is accessible in the manner that promotes in vitro METTL3/14 activity (Meiser et al., *Biol. Chem*, 2020). Since the only difference between our WT and A783U HOTAIR *in vitro* transcripts is the single A-to-U mutation at nucleotide 783, and therefore that is the only nucleotide that cannot be m6A modified in the mutant context, the changes we observe are most-likely due to lack of methylation. Observing disruption of YTHDC1 binding in the methylated A783U mutant (Figure 2C), along with the evidence above that A783 is available for methylation in the WT, supports this interpretation. We have added a brief description to clarify our logic in the text (Lines 213-215).

4. For the m6A and YTHDC1 RIP experiments, the authors refer to the other m6A sites in HOTAIR that are modified in MDA-MB-231 cells for the lack of difference between WT and mutant. How about MCF7 cells where the authors said that they only found a single site?

Upon additional analysis of the YTHDC1 RIP experiments in MDA-MB-231 cells, we observed a significant change in WT and A783U pulldown, where YTHDC1 RIP recovers significantly reduced levels of A783U HOTAIR compared to WT HOTAIR (Figure 2C). There is still a significant portion that is recovered, likely due to the other m6A sites in A783U HOTAIR. We have not performed genetic manipulation of the endogenous HOTAIR in MCF-7 cells to test for loss of YTHDC1 binding. However, to address this question further, we performed YTHDC1 RIP experiments in MCF-7 cells using qPCR oligos spanning different regions of HOTAIR and found that YTHDC1 specifically interacts with the region containing A783 (Figure 2D) in this context where A783 is the only detected methylated site.

5. Tethering YTHDC1 to A783U HOTAIR is a neat experiment. How does it affect cell growth and invasion when the need for m6A is bypassed this way? If the authors can see direct effects, that would strengthen the presented model.

We have performed analyses on doubling time and invasion upon tethering YTHDC1 to A783U HOTAIR and have added these data to Figure 6. We observed a decrease in doubling time (Figure 6E) and significant increase in invasion (Figure 6F) when YTHDC1 is tethered to A783U mutant HOTAIR. As noted in Point 6 response to Reviewer 1, these cancer cell biology experiments prove more challenging with the transient transection protocol for introducing the CasRx-tagged YTHDC1, which likely explains the milder effects than seen with molecular assays.

6. In multiple figures, the legend does not indicate what kind of values are being reported. Please state whether it is a mean or median, from how many replicates. Also, p values should always be indicated with what kind of statistical test.

We appreciate and agree with this reviewer's feedback. Values reported are stated in the Y-axis of each graph, and we have added information about number of replicates to each figure legend. Statistical methods are described in the Methods section, including information on box and whiskers and violin plots, as well as statistical test used to determine significance.

7. How do the authors reconcile the difference between Fig 2E and 3F? Is YTHDC1 level important or not for WT HOTAIR?

We thank the reviewer for this observation and note that reviewer #1 had a similar question (Point #3). While YTHDC1 levels do not induce significant changes in doubling time with WT HOTAIR, we note there is a trend towards shorter doubling time with more YTHDC1 (Fig 2E). However, the relationship between levels of HOTAIR and YTHDC1 in breast cancer patient data is complicated by mixed concordance of mRNA and protein levels for YTHDC1 (see Reviewer 1 Point #3 response for more detail). We have removed much of the YTHDC1 clinical data from the main text and consolidated a subset of it to Supplementary Figure 3.

8. In Fig 4B, how did the authors ensure that the amount of bait RNA is similar for all samples?

Both before and after methylation, RNA was isolated and quantified as described in the methods and equal amounts were then used for the experiments (Lines 797, 800, 820).

9. Can the authors distinguish between direct and indirect targets of HOTAIR for transcriptional regulation?

To attempt to distinguish between direct and indirect targets of HOTAIR that display the antimorph behavior, we examined HOTAIR-dependent H3K27me3 peaks identified by Meredith *et al.* (RNA 2016) for overlap with the differentially expressed genes we identified. A majority of the genes (76.5%) that are differentially expressed by WT HOTAIR compared to control and gain H3K27me3 peaks with WT HOTAIR overexpression also display antimorph behavior. Interestingly, these include the genes that are transcriptionally downregulated by HOTAIR overexpression, such as TP53I11 and SIRPA (Fig. 5B) and those that are upregulated (Fig. 5C). This helps inform how a repressive lncRNA can cause upregulation of some genes, likely through repression of a repressor locus near that gene. By examining the sites of HOTAIR genomic occupancy generated by Chu *et al.* (Mol Cell 2011) for overlap with HOTAIR v Control differentially expressed genes, we further identified 20 genes, 65% of which displayed an antimorph behavior, including a HOTAIR ChIRP peak in SEMA5A, another of our example HOTAIR-repressed, antimorph-sensitive genes (Fig. 5B). There are likely some indirect gene regulatory events occurring as well, but it appears that the majority of the genes that intersect in this antimorph mechanism are direct targets of the repressive function of HOTAIR.

Overall, this analysis reveals that a subset of the gene expression changes observed are likely to be directly regulated by HOTAIR chromatin interactions, while other changes are likely to be due to indirect effects. We have added a brief description of these analyses in the results (Lines 358-365) and discussion (Lines 477-482).

Reviewer #3: The authors study m6A on the lncRNA HOTAIR in the context of breast cancer. They performed an enormous amount of work using a range of techniques and obtain some interesting findings. However, several questions withing the study remain unresolved, and the proposed mechanism is somewhat convoluted.

1. If HOTAIR is highly expressed in TNBC patients, why do MDA-MB-231 (or MCF-7) cells not have high levels? While the authors are trying to make a case for their choice of model system, using ectopic expression of HOTAIR in 231 cells, I remain skeptical that this is indeed reflective of the in vivo situation in patients, in particular as m6A status in patients has not been studied here. A breast cancer cell line (and/or primary cells) with endogenously high HOTAIR levels may be a better model system. Replication of some key data in more clinically relevant cells or patient tissue could substantially elevate the findings.

We have screened additional cell lines recently generated from patient-derived xenografts (Finlay-Schultz *et al.* (Breast Cancer Research 2020)) for HOTAIR levels and m6A sites. We identified m6A783 in one of these lines, supporting the potential that methylation of this site can occur in patient tumors. In general, the other lines expressed levels of HOTAIR that were likely too low for easy m6A detection. Overall, the exciting result that we identify this site, and this site alone, in recently derived cell lines from breast tumors, supports the potential for it to be influential in a breast tumor context.

2. Figure 1D does not have a "yellow to red scale" as indicated in the legend, it seems like the color scheme has been changed without updating the legend

We thank this reviewer for noting this error. The legend has now been updated.

3. Why does endogenous HOTAIR only have one m6A site in MCF7 while overexpressed HOTAIR has 8 m6A sites? Is this dependent on expression level or cell line or BC subtype?

To address this question, we have mapped m6A sites in additional breast cancer cell lines as noted in the response to issue 1. The results suggest that the prevalence of m6A in HOTAIR scale with expression level: m6A was only detected in HOTAIR where it was most highly expressed, and only at A783 in a subset of samples. However, we cannot rule out other contexts that influence the methylation at this time. We have addressed this briefly in the discussion (Lines 437-440).

4. Phenotypic differences between cells transfected with HOTAIR and the A783U mutant seem rather modest overall, and there is no evidence that this is reflective of a patient tumour.

We appreciate the reviewer's comment. While using cell lines is certainly far removed from a patient tumor and we do not mean to conclude a direct relationship, we propose that our results are potentially suggestive of patient tumor biology, especially considering the UCD4 cell line, recently derived from a patient, that we found has HOTAIR methylation at A783. We have added a passage to the discussion to note this potential, along with the limitations of mainly using long-standing cell lines in our work, in the Discussion (Lines 460-462).

5. The authors switch back and forth between the two cell lines, while unclear if findings in one also hold up in the other. Is there a phenotypic effect on cell growth in MCF-7 as well? Does hnRNP B1 bind HOTAIR m6A783 in MDA-MB-231 cells? Does YTHDC1 oe/kd impact on growth in MCF7 cells?

We are happy to clarify the use of each of these cell lines. Each cell line has its own advantages for asking different questions: MCF-7 cells that have only a single m6A site detected in HOTAIR and express endogenous HOTAIR at low levels are useful for some experiments that address the specific interaction at A783 without any manipulation. MDA-MB-231 cells that do not express detectable levels of endogenous HOTAIR are useful for analysis of transgenic overexpression of WT or A783U HOTAIR and are meant as a mimic of the context in breast cancer tumors with high HOTAIR.

6. How do WT / control transfected MDA-MB-231 behave when YTHDC1 is overexpressed or knocked down? HOTAIR won't be the only target, and the effects may be indirect on target genes of HOTAIR.

We agree with this reviewer that there are possible pleiotropic effects of YTHDC1 overexpression and knockdown. This is noted in the text (Lines 246-248, 394-397). With the advantages of the more specific strategy of mutating A783 and then tethering YTHDC1 directly to HOTAIR (see Figure 6), we are able to focus our analysis to the functions of YTHDC1 association with A783 specifically.

7. Correlation between YTHDC1 and HOTAIR in patients may be "very modest" due to the fact that the two genes simply don't correlate. The survival curves are all indirect measures, and the patient data is overall somewhat hard to interpret in the context of this story as patients' m6A status is unknown.

We thank this reviewer for noting this concern and have now removed a large amount of the clinical data looking at YTHDC1 mRNA levels, as we note that issues with lack of concordance between YTHDC1 mRNA and protein levels make analysis of these clinical samples less robust.

8. Since binding of YTHDC1 does not change whether the A783U mutant is methylated or not, it is questionable whether a) this residue is really as important as the rest of the manuscript suggests and b) whether interactions with YTHDC1 are mediated by A783me at all. A bit confusing in light of the difference in cell doubling data and the difference in chromatin association. The given explanation regarding highly constitutive interactions and binding sites of different affinities that change in the mutant seem all rather speculative. What happens to breast cancer cells when the other m6A residues are mutated?

We thank the reviewer for highlighting where the manuscript needed additional support of the conclusions. In response to this and other reviewers' concerns, we have more extensively analyzed binding of YTHDC1 to HOTAIR. We found that RNA immunoprecipitation of YTHDC1 results in increased recovery of the region of HOTAIR that contains A783 (Figure 2D) and decreased recovery of YTHDC1 in the HOTAIR A783U mutant (Figure 2C). We know that many m6A sites remain in the absence of A783 methylation when HOTAIR is at high levels in the A783U mutant, including one site just upstream of A783, explaining how YTHDC1 IP still recovers HOTAIR.

To further address the cell biology of mutants of the other sites, we have also analyzed the doubling time for MDA-MB-231 cells expressing the 6xAU and 14xAU mutations and found that cells display a doubling time similar to control Anti-Luciferase expressing cells (Supplementary Figure 4D). This is consistent with our observation that removal of the other m6A sites causes a loss in HOTAIR levels, suggesting that these sites promote stability of the RNA. Without these methylation events, MDA-MB-231 cells behave similar to those without any HOTAIR transgene.
